# Bipolar ionization rapidly inactivates real-world, airborne concentrations of infective respiratory viruses

**DOI:** 10.1371/journal.pone.0293504

**Published:** 2023-11-22

**Authors:** Edward Sobek, Dwayne A. Elias

**Affiliations:** 1 Global Plasma Solutions, Charlotte, NC, United States of America; 2 Elias Consulting, LLC, Knoxville, TN, United States of America; Satyawati College, University of Delhi, INDIA

## Abstract

The SARS-CoV-2 (COVID-19) pandemic has highlighted the urgent need for strategies that rapidly inactivate airborne respiratory viruses and break the transmission cycle of indoor spaces. Air ions can reduce viable bacteria, mold, and virus counts, however, most studies use small test enclosures with target microbes and ion sources in close vicinity. To evaluate ion performance in real-world spaces, experiments were conducted in a large, room-size BSL-3 Chamber. Negative and positive ions were delivered simultaneously using a commercially available bipolar air ion device. The device housed Needle Point Bipolar ionization (NPBI) technology. Large chamber studies often use unrealistically high virus concentrations to ensure measurable virus is present at the trial end. However, excessively high viral concentrations bias air cleaning devices towards underperformance. Hence, devices that provide a substantial impact for protecting occupants in real-world spaces with real-world virus concentrations are often dismissed as poor performers. Herein, both real-world and excessive virus concentrations were studied using Influenza A and B, Human Respiratory Syncytial Virus (RSV), and the SARS-CoV-2 Alpha and Delta strains. The average ion concentrations ranged from 4,100 to 24,000 per polarity over 60-minute and 30-minute time trials. The reduction rate was considerably greater for trials that used real-world virus concentrations, reducing infectivity for Influenza A and B, RSV, and SARS-CoV-2 Delta by 88.3–99.98% in 30 minutes, whereas trials using in-excess concentrations showed 49.5–61.2% in 30 minutes. These findings strongly support the addition of NPBI ion technology to building management strategies aimed to protect occupants from contracting and spreading infective respiratory viruses indoors.

## Introduction

Prior to the 2019 Covid pandemic, the Centers for Disease Control and World Health Organization recognized droplet and fomite transmission as primary modes of host infection [1]. New hosts were primarily infected though the nose, eyes, and mouth directly by droplets or less so indirectly by fomite transmission when a potential host touched a surface harboring droplets [2–4]. Infectious droplets typically travel one to two meters before settling onto surfaces, so to reduce transmission rates, health agencies implemented social distancing [5]. However, data collected on SARS-CoV-2 transmission forced agencies to consider the new paradigm of droplet nuclei transmission in which expelled droplets rapidly evaporate and release active virus particles that become airborne for an extended period time before infecting a host [6]. Droplet nuclei are not limited to sneezing and coughing, but are released during resting exhalation and vigorous exhalation related to exercise, singing, and shouting [7], forcing health agencies to reconsider prevention methods such as masking and implementation of air cleaning technologies that remove or inactive respiratory viruses.

The goal of this study was to assess NPBI, a soft bipolar ionization technology, as an effective tool for inactivating respiratory viruses transmitted indoors via droplet nuclei. Bipolar ionization is effective at agglomerating ultrafine particles [8–10], including viruses which then fall onto surfaces. This technology was certified as free of generating Ozone by United Laboratories (Zero Ozone Emissions Validation | UL Solutions). For this study, the term “indoor” includes enclosed spaces such as houses, apartments, workplaces, schools, commercial units, airports, and hospitals [11]. The data presented herein were obtained using carefully conducted experiments to better understand the parameters associated with virus inactivation by NPBI. All experiments were conducted within a sealed testing room approved for safe handling of Biosafety Level-3 (BSL-3) microorganisms. Rather than simply testing one virus with one device, we report the effect of NPBI ionization on Influenza A, Influenza B, RSV, and the SARS-COV-2 Alpha and Delta variants. A range of ion levels were evaluated to determine the effect that ion density (ions/cc air) has on virus infectivity over time. Lastly, we compared the rate of NPBI virus inactivation with artificially high virus titers (commonly used in laboratory efficacy testing) to real-world virus titers that have been quantified within indoor spaces [12].

## Methods

### Viral strains used

The strain of SARS-CoV2 used was SARS-COV-2 USA-CA1/2020 and was obtained through BEI Resources, NIAID, NIH: SARS-Related Coronavirus 2, Isolate USA-CA1/2020, NR-52382. The SARS-CoV-2 Delta Variant was obtained through BEI Resources, NIAID, NIH: SARS-Related Coronavirus 2, Isolate hCoV-19/USA/PHC658/2021 (Lineage B.1.617.2; Delta Variant), NR-55611, contributed by Dr. Richard Webby and Dr. Anami Patel. The Respiratory Syncytial Virus (RSV) was obtained through BEI Resources, NIAID, NIH: Human Respiratory Syncytial Virus, A2001/2-20, NR-28525. The Influenza A Virus (NR-31132) was obtained through BEI Resources, NIAID, NIH: Influenza A Virus, A/mallard/Wisconsin/2785/2009 (H2N3), NR-31132. The Influenza B Virus was obtained through BEI Resources, NIAID, NIH: Influenza B Virus, B/New York/1055/2003, NR-48660. Quality control data is listed in S1-S5 Tables in S1 File.

### Viral sample preparation

The most common method for studying virus viability is the Tissue Culture Infectious Dose 50 assay, or TCID50 which quantifies viral titer by determining the concentration at which 50% of the infected cells display a cytopathic effect (CPE) including changes in cell shape, fusion with surrounding cells, or inclusion body appearance in host cells. As long as the virus being studied results in cell death, this assay can be used when viral antibodies are not available, and little information on the virus is required. Serial dilutions of virus were plated onto VeroE6 host cell monolayers, left for 24 hours, and scored to find where 50% of host cells displayed CPE for quantitation of the original virus concentration; i.e. a TCID50. To calculate the actual amount of virus per ml, the TCID50 is multiplied by 0.7 according to the Poisson distribution [13]. Once accomplished, this concentration was used to assess the NPBI technology for degrading viral viability.

One day before the Vero E6 cell host infection (Vero C1008, ATCC No. CRL-1586, a line cloned from Vero 76), 96 well plates were seeded with the Vero E6 cells [14] obtained from the American Tissue Culture Collection (ATCC; https://www.atcc.org/products/crl-1586), prepared in Dulbecco’s Modified Eagle Medium (DMEM) plus fetal bovine serum, 4mM Glutamine, and antibiotics, and incubated at 32°C overnight. Viral samples were serially diluted 1:10 (v:v) in Phosphate buffered Saline (PBS). The Vero E6 cells (0.1 mL) were mixed with 0.1 mL of each viral dilution in quadruplicate, and the virus allowed to absorb to the cells at 37°C for 2 hours. After absorption, 0.5 mL was added to each well and incubated at 37°C with the CPE monitored using an inverted microscope over one to four weeks with the number of positive and negative wells recorded. The dilution that caused a 50% infectivity rate of host cells was used in this study as the Tissue Culture Infectious Dose (TCID 50/mL) [13]. Once the desired TCID50/mL was determined, 30 mL of the sample was prepared with 10 mL used for each replicate control or test trial so as to begin with the most similar starting concentration.

### Ionization technology to be tested

**Fig 1:**
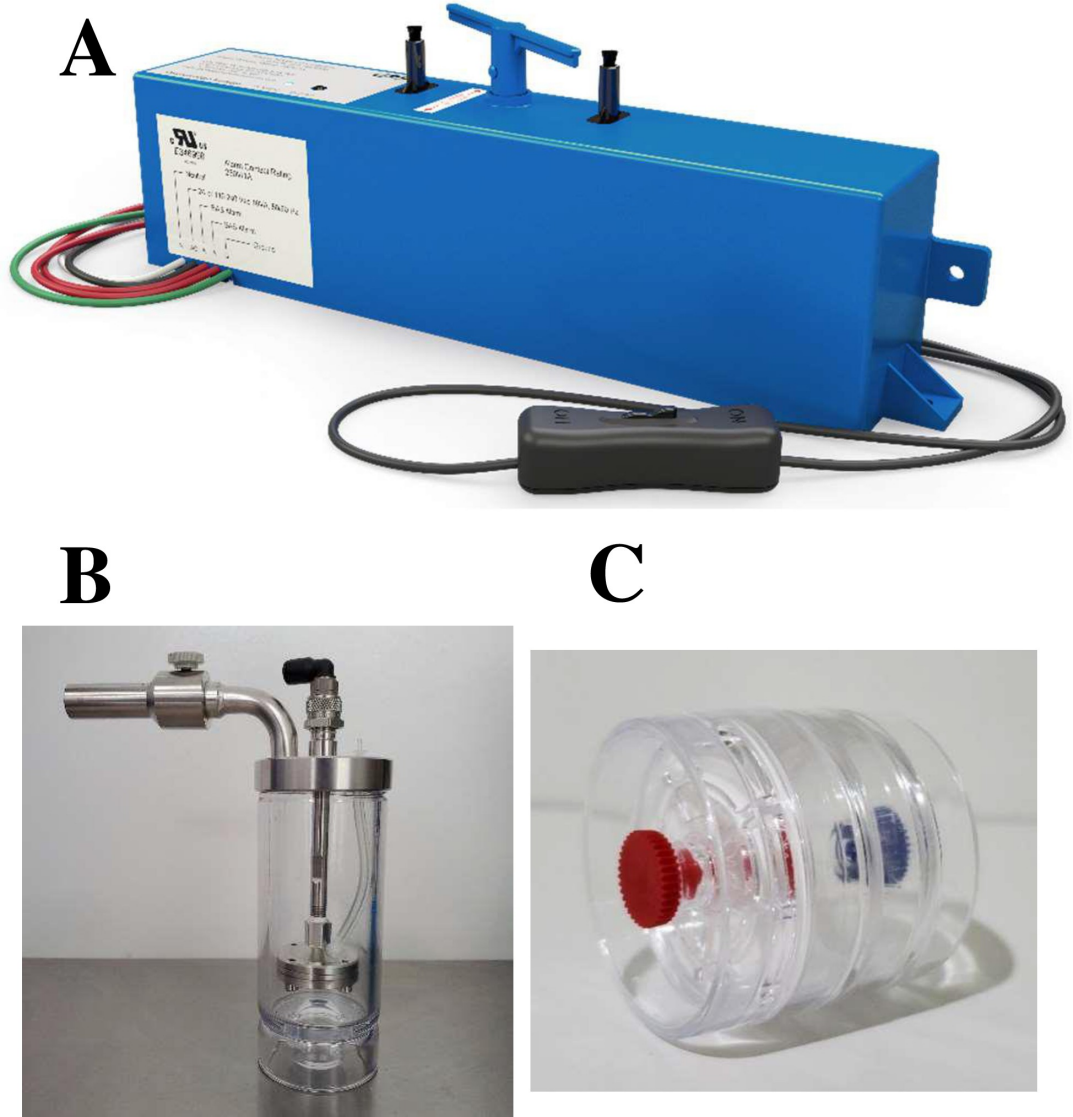
Equipment used in study. The GPS-FC48-AC™ ionizing device (A), the BLAM nebulizer (B), and the Sensidyne air sampler cassette (C).

The GPS-FC48-AC (Fig 1A) manufactured by Global Plasma Solutions (GPS; https://gpsair.com/) was used to test NPBI against all viruses listed. NPBI is a soft ionization technology that incorporates emitters comprised of bundled microfiber carbon brushes. Large radius brush emitters ensure constant low voltage operation over the lifetime of the device. In contrast, steel needles erode and dull over time, leading to arcing and voltage fluctuations, requiring replacement at regular intervals. NPBI was designed for devices that are installed in typical HVAC systems or drop-ceiling destratification units. The technology produces an ion-rich cloud for duct distribution or ceiling distribution into building spaces. Soft ionization technology was initial developed as a toxicological tool to identify intact molecules in the 1970’s [15]. Unlike hard ionization which uses a high energy electron beam that can fragment molecules into an atomic mass fingerprint for identification, soft ionization only adds an electron or removes an electron from the molecule. The target molecule remains intact with a net charge. Common ions include the core atmospheric gases in the form of hydronium ion clusters O_2_^-^, CO_4_^-^(H_2_O)_n_, and NO_3_^-^(H_2_O)_n_ and are described in detail as the lower E e^-^ ion-molecule interactions for water clusters Y^-^(H_2_O)_n_ by Sekimoto and Takayama [16]. The development of soft ionization allowed for identification of plant and fungal derived poisons and many other biological compounds and environmental toxins.

**Fig 2:**
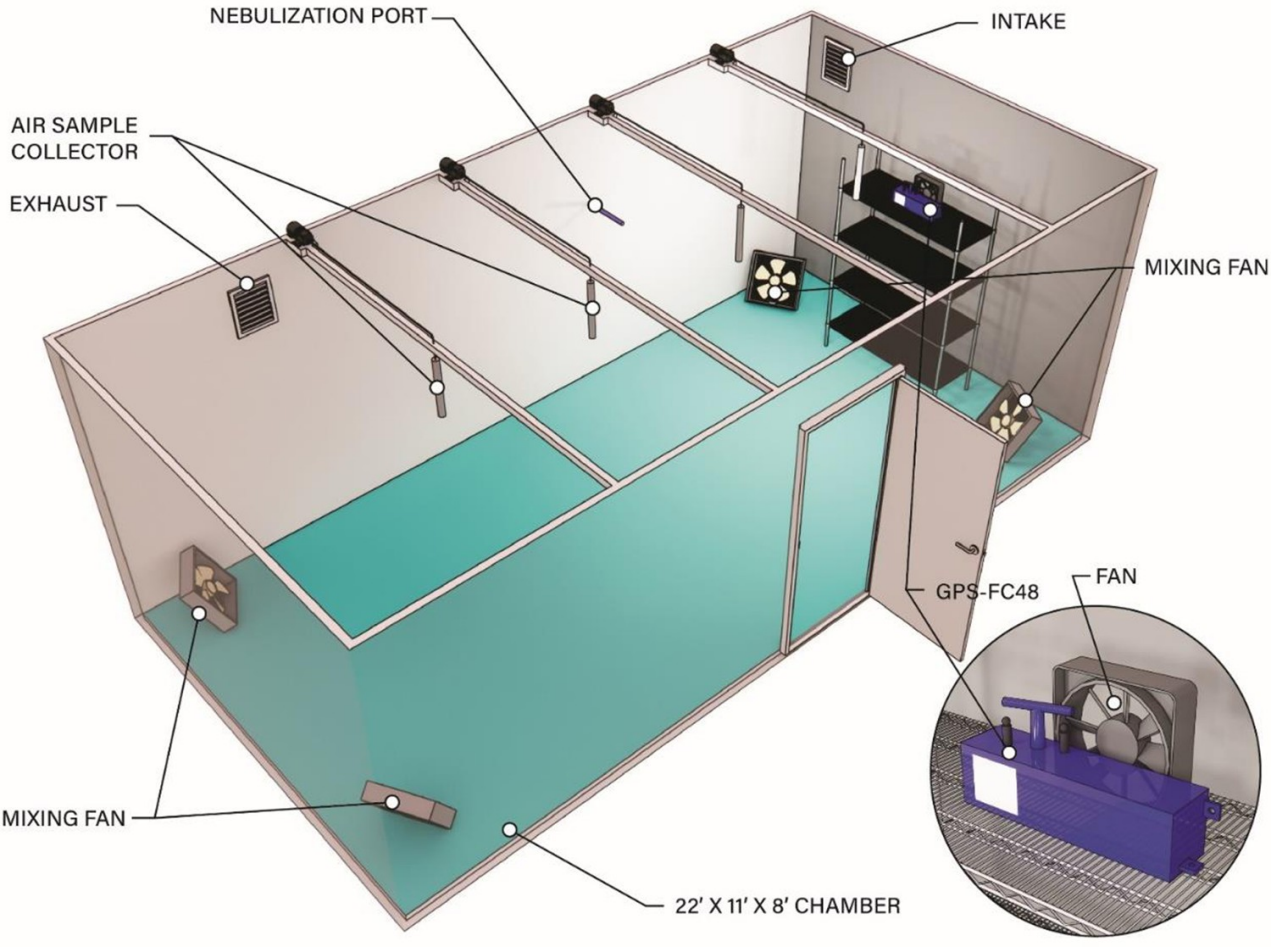
Room schematic. A schematic of the test room with placement of the GPS-FC48-AC™ device.

Two Alpha Lab, Inc. (https://www.alphalabinc.com/, West Salt Lake City, UT, USA) AIC2 ion polarity Gerdien meters with USB datalogging confirmed negative and positive ion generation. The variable speed fan located behind the NPBI device (Fig 2) was adjusted to obtain the average room ion density for each test while measuring ion values at multiple points in the room. Average room ion density ranged from 4,100 ions/ cm^3^ to 15,790 ions/ cm^3^ positive ions and 4,900 ions/ cm^3^to 24,070 ions/ cm^3^ negative ions (Table 1) depending upon the test being conducted. Due to the nature of ions, there were fluctuations in concentrations around the entire room.

**Table 1:** Ions levels used in all trials with the average loss of virus activity over time.

TEST																	
Virus tested	Positive ion level/cm^3^	Negative ion level/cm^3^	Virus Particles/L 0 min	Virus Particles/L 30 min	Virus Particles/L 60 min		Gross Reduction Rate 30 min	Gross Reduction Rate 60 min		Net Reduction Rate 30 min	Net Reduction Rate 60 min		Rate of Reduction 30 min	Rate of Reduction 60 min		Percent improvement 30 min	Percent improvement 60 min
Influenza A	11,930	22,830	4,966	2,139	7		56.9%	99.9%		44.7%	99.8%		-94.2	-82.7		157.4%	143.2%
Influenza B	12,900	22,850	577	297	51		48.5%	91.2%		34.4%	82.8%		-9.3	-8.8		124.8%	87.4%
RSV	9,600	23,600	654	313	9		52.1%	98.6%		36.0%	97.2%		-11.4	-10.8		106.0%	92.4%
SARS-CoV-2	4,100	4,900	152	0			99.9%			99.7%			-5.1			31.5%	
SARS-CoV-2	21,000	12,000	170	0			100.0%			100.0%			-5.7			45.9%	
SARS-CoV-2	9,400	18,600	157	0			100.0%			100.0%			-5.2			42.9%	
SARS-CoV-2 Delta	15,790	24,070	3,894	1,564	0		59.8%	100.0%		45.8%	100.0%		-77.7	-64.9		130.9%	48.0%
CONTROL																	
Virus tested	Positive ion level/cm^3^	Negative ion level/cm^3^	Virus/L 0 min	Virus/L 30 min	Virus/L 60 min		Gross Reduction Rate 30 min	Gross Reduction Rate 60 min					Rate of Reduction 30 min	Rate of Reduction 60 min			
Influenza A			4,966	3,868	2,927		22.1%	41.1%					-36.6	-34.0			
Influenza B			577	452	296		21.6%	48.6%					-4.2	-4.7			
RSV			654	489	319		25.3%	51.2%					-5.5	-5.6			
SARS-CoV-2			152	36			76.1%						-3.9				
SARS-CoV-2			170	54			68.4%						-3.9				
SARS-CoV-2			157	47			69.9%						-3.7				
SARS-CoV-2 Delta			3,894	2,885	1,264		25.9%	67.5%					-33.6	-43.8			

### Bioaerosolization

Nebulization was accomplished using a Blaustein Atomizing Module (BLAM) from CH Technologies USA (https://chtechusa.com/products_tag_lg_blaustein-atomizing-modules-blam.php, Fig 1B) with a pre-set PSI and computer-controlled liquid delivery system. Before testing, the solution was nebulized without virus to confirm average particle size distribution. For all trials, after nebulization the BLAM’s remaining volume was weighed before and after each trial to confirm that a similar amount was nebulized. Bioaerosol procedures for the controls and viral tests were performed in the same manner with corresponding time points and collection rates in triplicate.

For each virus, 10 mL of the TCID50/mL was used and nebulized at 1mL/min. For trials with SARS-CoV-2, the nebulizer was filled with 9.63 x 10^5^ TCID50/mL, for the Delta variant, 2.47 x 10^7^ TCID50/mL, 3.15 x 10^7^ TCID50/mL of Influenza A, 3.66 x 10^6^ TCID50/mL of Influenza B, and for RSV, 4.15 x 10^6^ TCID50/mL. All test trials were conducted at ~10,000–15,000 positive ions/cm^3^ and ~21,000–24,070 negative ions/cm^3^ to compare the relative efficiency of viral removal via agglomeration as detailed below. For SARS-CoV-2, we chose to test NPBI at various ion concentrations by varying the air flow rate, so as to determine the efficiency of viral removal at the lower ion concentrations.

### Bioaerosol sampling

Four Sensidyne 37mm directional air flow sample cassettes were used (Fig 1C) for sampling with each connected to a calibrated Gilian 10i vacuum device and set at 5.02L/min with a 0.20% tolerance. The sample collectors were suspended from the ceiling at increasing distances from the ion generator (Fig 2). Before use, the vacuum system calibration was confirmed using a Gilian Gilibrator-2 NIOSH Primary Standard Air Flow Calibrator (Sensidyne, LP, St. Petersburg, FL). Air sample collections occurred over either 60-minute or 30-minute durations. Sample collection volumes were set to 10-minute draws, allowing for ~50 L of air collection per port. The air sampler internal filtration discs were moistened with virus media prior to the test to aid in collection. Filtration discs from Zefon International, Lot# 26338, were used for testing. At each time point, all sample discs were pooled for an average across the four sampling locations.

### Room description

Testing was conducted in a sealed 20’x8’x8’ (6.1x2.4x2.4m) BSL-3 chamber. The overall dimensions provided a total air volume of 36,245.6 L. A nebulizing port with a programmable compressor in the center of the 20-ft wall protruded 24 inches (Fig 2). At each corner, low-volume mixing fans, ~30 ft^3^/min (0.85m^3^) each, were positioned at 45-degree angles for homogenous mixing. For sampling, four probes were positioned along the centerline and protruded down 24” (0.61 m).

A GPS-FC48-AC ionizer was in the room’s centerline and elevated 6ft (1.8m; Fig 2). During testing, ion measurements were taken directly above the samples for consistent readings. A variable-speed fan behind the GPS-FC48-AC created the necessary airflow to produce ions. The chamber was visually inspected, pressure tested, and all internal lab systems and equipment were reviewed before testing. The temperature during testing was 73 ± 2°F (22.8±1°C), with a relative humidity of 40 ± 2%.

### Chamber control and test conditions

Control tests were conducted in triplicate with the fan turned on and the device present as in the test trials but turned off. The BLAM was loaded with 10 mL of viral suspension and operated as described above. Samples were taken at 0, 15, 30, 45, and 60 minutes. The control results showed a natural rate of loss and were used to assess the ability of the NPBI technology to reduce SARS-CoV-2 in the air.

Before and after each trial, the UV system inside the testing chamber was activated for 30 minutes, followed by a 30-minute HEPA air purge. All test equipment was cleaned each day with 70% isopropanol. Collection lines were soaked in bleach for 30 minutes and rinsed repeatedly with distilled water. The nebulizer and vacuum collection pumps were decontaminated with hydrogen peroxide.

Once the room was cleaned and baseline/background air measurements completed, 10 mL of virus in viral media was nebulized into the room via the dissemination port (Fig 2). The completion of nebulization was marked as time 0 (T = 0) and the GPS-FC48-AC was turned on via remote control. The device was turned off at each sample collection of 10 minutes so as to not further degrade the virus during the sampling, and obtain a more uniform sampling from the indicated time for each replicate. Sample cassettes were manually removed and taken to an adjacent biosafety cabinet to be physically pooled into one collection tube per time point, providing an average across the four sampling locations. All samples were sealed for analysis after the study completion. Each of the pooled samples for each time point in each trial was used as the inoculum for another TCID50/mL assay in order to determine titer of the virus remaining in each pooled sample. Once calculated, these data were used to generate the figures and tables.

### Data analysis

Data analysis used averages and standard deviations calculated from replicates. Rates of viable virus loss were calculated by subtracting the TCID50/mL or the calculated viable virus/L of room air of a later time point from the earlier time point and dividing by the elapsed minutes (eg. T_30_-T_0_/30). While all TCID50/mL data is in the supplemental, we calculated the viable virus particles/L of room air to present the data in a more relatable fashion; Eq (1). Since Sensidyne collection filters have a 65–70% efficacy for capturing particles [17, 18], that was built into the calculations:

Viablevirus/Lroomair=(((C1*1000mL/L)*V1)/V2)/E (1)

Where:

C1 = the TCID50/mL value.

V1 = the BLAM volume of 10 mL (0.01L).

V2 = the testing room air volume of 20’x8’x8’ or 1,280 ft^3^ (36,245.6 L).

E = collection filters 70% efficacy for capturing particles.

The extent of ion effects on viral viability was calculated using Gross Virus Reduction Rate, the Net Virus Reduction Rate, the Overall Virus Rate of Reduction, and the Percent Improvement. The Gross Virus Reduction Rate (GVRR) is a percent difference in virus infectivity for each sample period versus the starting virus concentration for a given decay curve calculated by subtracting the virus concentration at a given time point from time zero concentration and then dividing by the time zero concentration (eg. (T_0_-T_30_/T_0_*100) for a given decay curve. The Net Virus Reduction Rate (NVRR) is the percent decrease in virus infectivity between the control decay and the experimental decay at the same time point to evaluate air cleaning device performance in decreasing airborne virus activity. The NVRR was calculated by subtracting the control virus concentration from the treatment virus concentration at a given time point and then dividing by the control virus concentration (eg. T30_control_-T30_treatment_/T30_control_). The Overall Virus Reduction Rate (OVRR) is the decrease in virus infectivity attributed to the air cleaning device per unit time and calculated by subtracting the elapsed time point virus concentration from the time zero virus concentration and dividing by the elapsed time in minutes (eg.T_0_-T_30_/30). Lastly, the Percent Improvement (PI) is a measure of the effectiveness of the device in removing the virus from the room air space, and was calculated by subtracting the treatment OVRR from the control OVRR, then dividing by the control OVRR and multiplying by 100 (eg. (((OVRR^T30^_treatment_-OVRR^T30^_control_)/OVRR^T30^_control_)*100).

All statistical analysis was completed using Minitab statistical software version 21.3.1 (https://www.minitab.com/). Because each exposure time trial was conducted separately for each replicate, exposure time was classified as an independent factor in the analyses. Hence, a general linear model 2-way analysis of variance (ANOVA) was conducted for each virus trial, where Exposure Time with multiple levels and NPBI with two levels (Test/Control) were classified as categorical factors, and virus concentrations was classified as the continuous response variable. The model consisted of Exposure Time, NPBI, and Exposure Time by NPBI interaction. Post-hoc analysis to identify statistical differences associated with exposure time and ion level was conducted using Tukey pairwise comparisons [19]. Since ANOVA is most sensitive to unequal variances, the Levene test for equivalence of variance [20] was ran before ANOVA. All groups across each trial were confirmed to have variance homogeneity (S10 Table in S1 File).

## Results

### Effects of virus titer and carrier solution on NPBI efficacy in the testing room

The current working hypothesis for viral inactivation by NPBI is that an abundance of positive and negative ions modify virus charge thereby disrupting the spike-protein trimer configuration, which is critical for virus attachment to host receptors [21].

**Fig 3:**
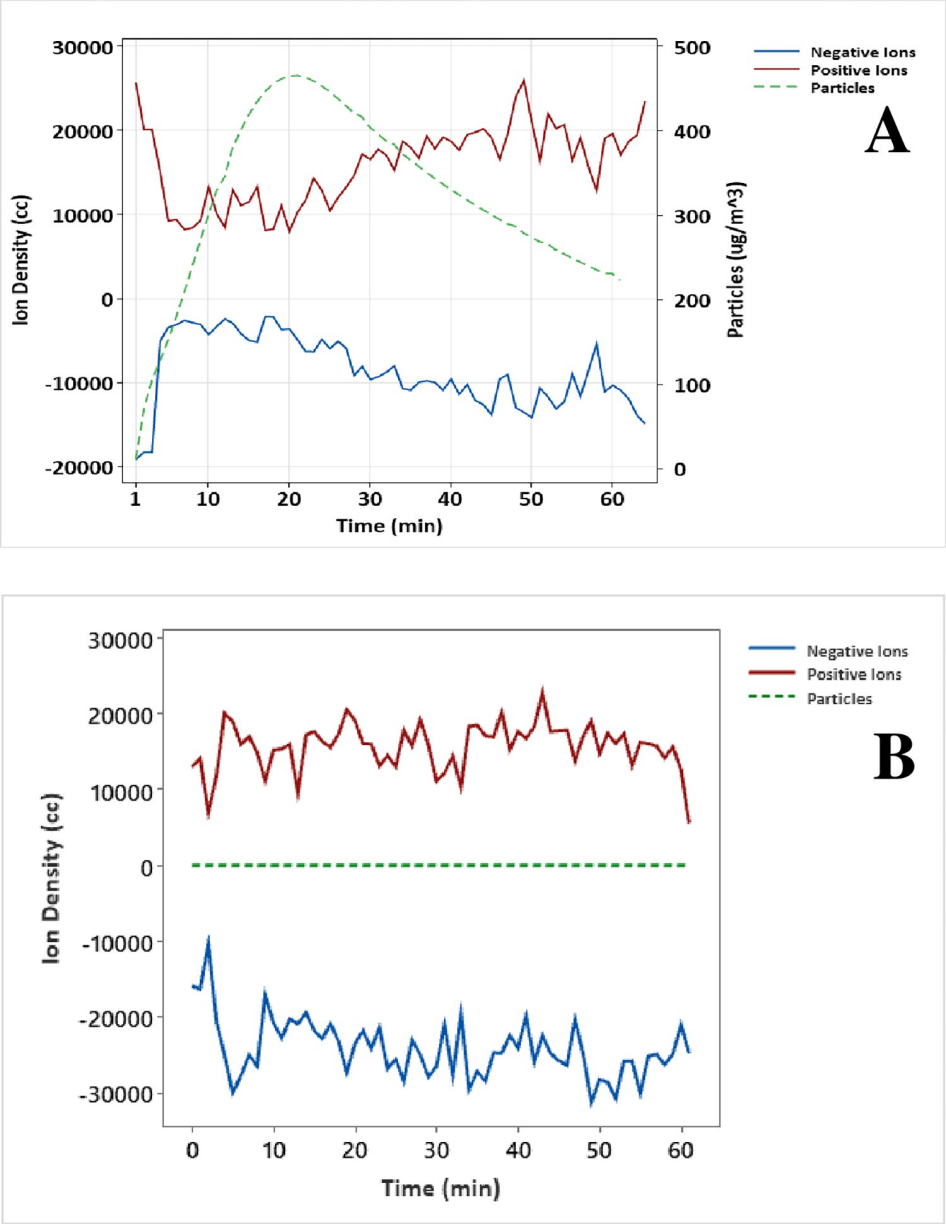
Ion suppression curve. Ion concentrations (positive red; negative blue) decreased rapidly following carrier nebulization (green) and suppression continued for 15 minutes until particle mass reached its asymptote, and then ions rebounded towards the pre-nebulization starting concentration for the remainder of the trial (A). In contrast, ion suppression was absent when no carrier or virus particles were injected, and ions rapidly increased to an average ion density of 25,000 ions/cc (B). The chamber rapidly filled with ions, but the mass of particles < 2.5 um remained near zero and constant for the duration of the study (~1.86 particles/μg).

The ion density and particle concentrations were recorded during the SARS-CoV-2 virus carrier trials (Fig 3) with temporal data for positive and negative ions as well as the small particle concentration (< 2.5 μm). At time zero, the room ion density was equal to the test setpoint. Upon virus carrier nebulization, the ions instantaneously attached to the ultrafine carrier particles with an aerodynamic diameter of 0.1 μm or less [22, 23] (Fig 3A). Ion output was static but as small particles increased during nebulization, free ion availability became limited, resulting in ion suppression at 1–2 minutes. Nebulization lasted five minutes as ultrafine particles increased and briefly plateaued. Bipolar ionization is effective at agglomerating ultrafine particles [8–10], and with no further input of ultrafine particles, particle size and mass increased to where inertia cause agglomerated particles to deposit onto surfaces. This caused the percentage of free ions to rebound, further increasing agglomeration and deposition rates as evident by the decreased particle concentration after 20-minutes until the trial end where the ions returned to the starting density. No such suppression effects were observed when the carrier solution was not nebulized into the testing room (Fig 3B).

### Control trials

For each virus tested, control trials were conducted in triplicate without the GPS-FC48-AC being activated. Virus preparation and nebulization occurred as detailed above with the 30 mL of virus preparation being divided into 10 mL aliquots for the triplicate trials. Samples were taken over the course of 30 or 60 minutes depending upon the virus used, where zero (“0”) minutes was the end of the nebulization period. The results were plotted to show a natural rate of loss and to demonstrate the high degree of similarity and repeatability between the control or test replicates. The rate and total difference in viral loss between the control and test trials were used to assess the NPBI’s ability to reduce the viral load in the room air.

**Fig 4:**
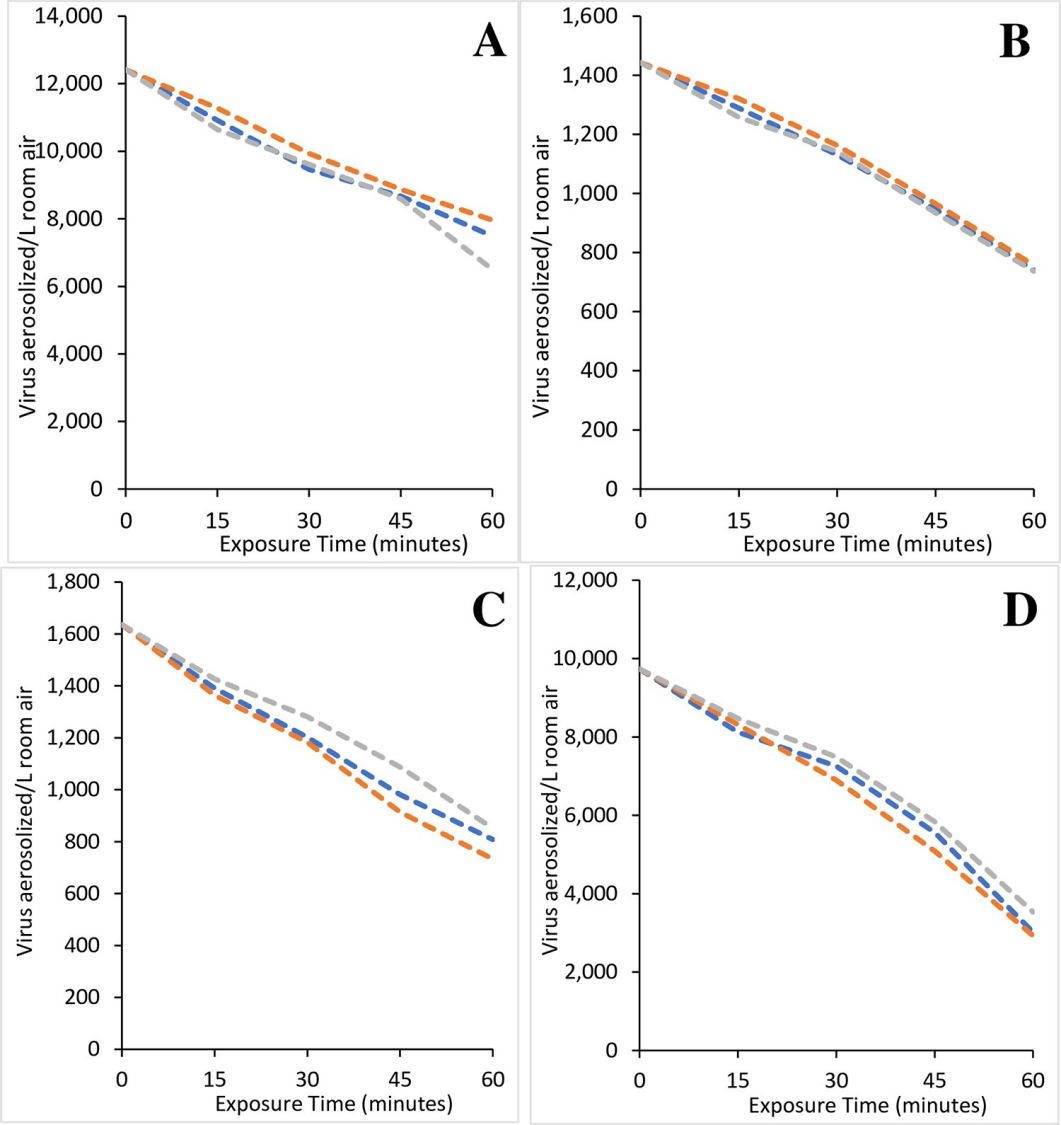
Triplicate control runs for viruses. Sixty-minute individual control runs to determine the natural loss of Influenza A (A), Influenza B (B), RSV (C), and the SARS-CoV-2 Delta variant (D). The individual trials demonstrate the similarity of the replicate trials and repeatability of the system used in this study.

**Fig 5:**
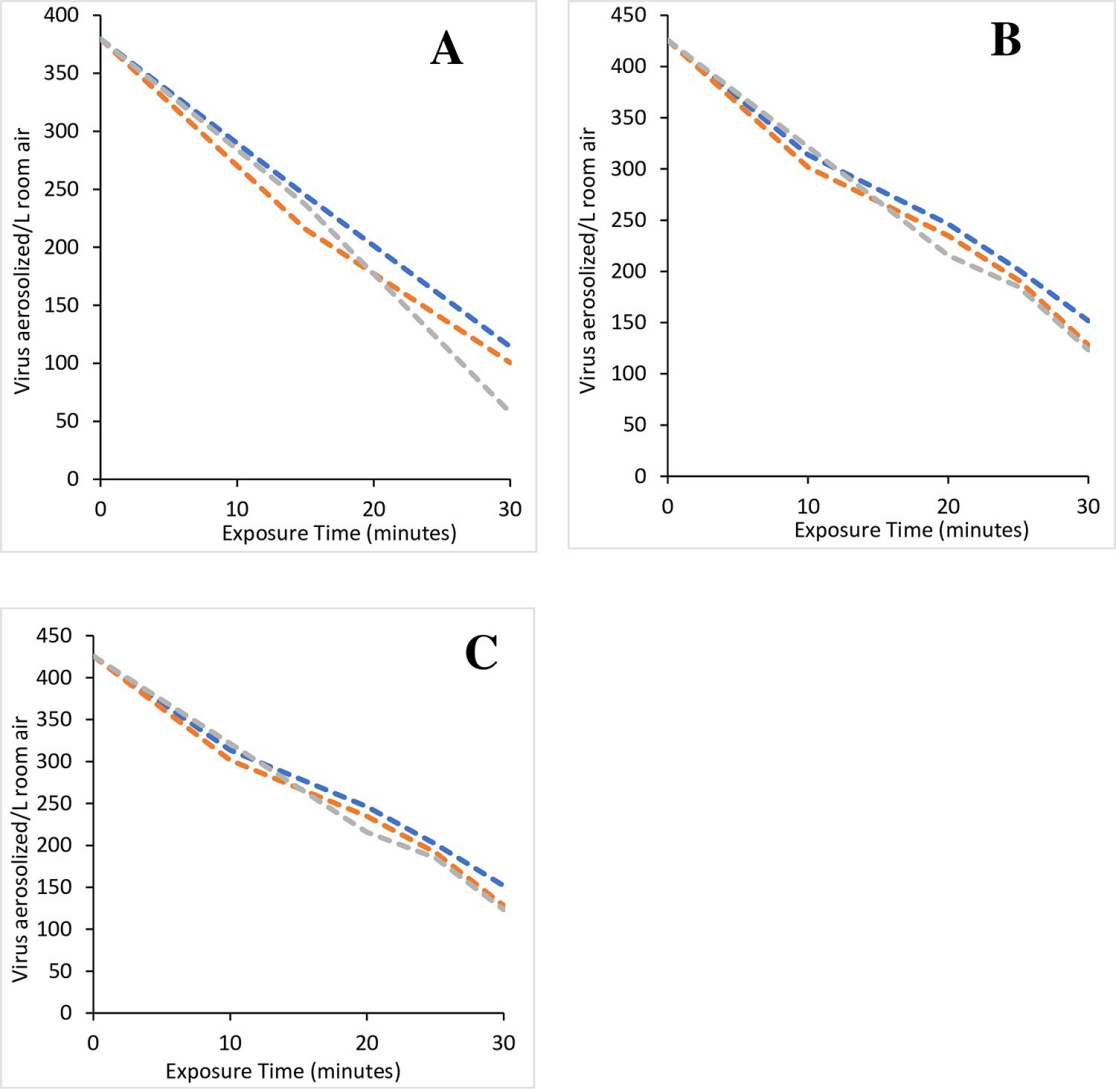
Triplicate control runs for SARS viruses. Thirty-minute individual control runs to determine the natural loss of SARS-CoV-2 to compare with the GPS-FC48-AC operating at 4,900 negative ions/cm^3^ (A), SARS-CoV-2 to compare with the GPS-FC48-AC operating at 12,000 negative ions/cm^3^ (B), and SARS-CoV-2 to compare with the GPS-FC48-AC operating at 18,000 negative ions/cm^3^ (C). The individual trials demonstrate the similarity of the replicate trials and repeatability of the system used in this study.

**Fig 6:**
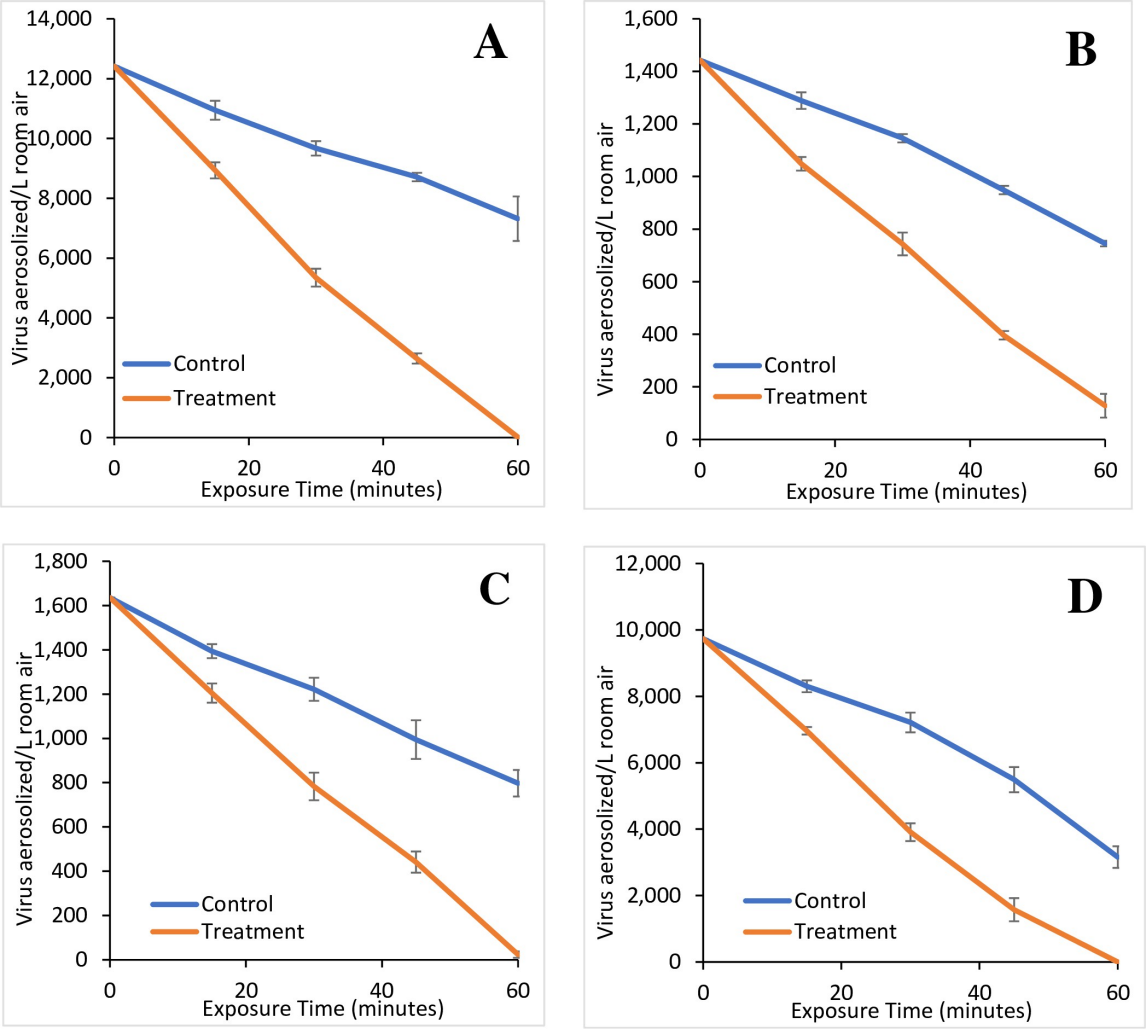
Virus loss with ion treatment. The average loss over time of Influenza A in the absence (control) and presence (treatment) of an operating GPS-FC48-AC (A), Influenza B in the absence (control) and presence (treatment) of an operating GPS-FC48-AC (B), RSV in the absence (control) and presence (treatment) of an operating GPS-FC48-AC (C), SARS-CoV-2 Delta variant in the absence (control) and presence (treatment) of an operating GPS-FC48-AC (D). Plots are the average of triplicates for the control and treatments. Error bars represent the standard deviation of the replicates at each time interval.

**Fig 7:**
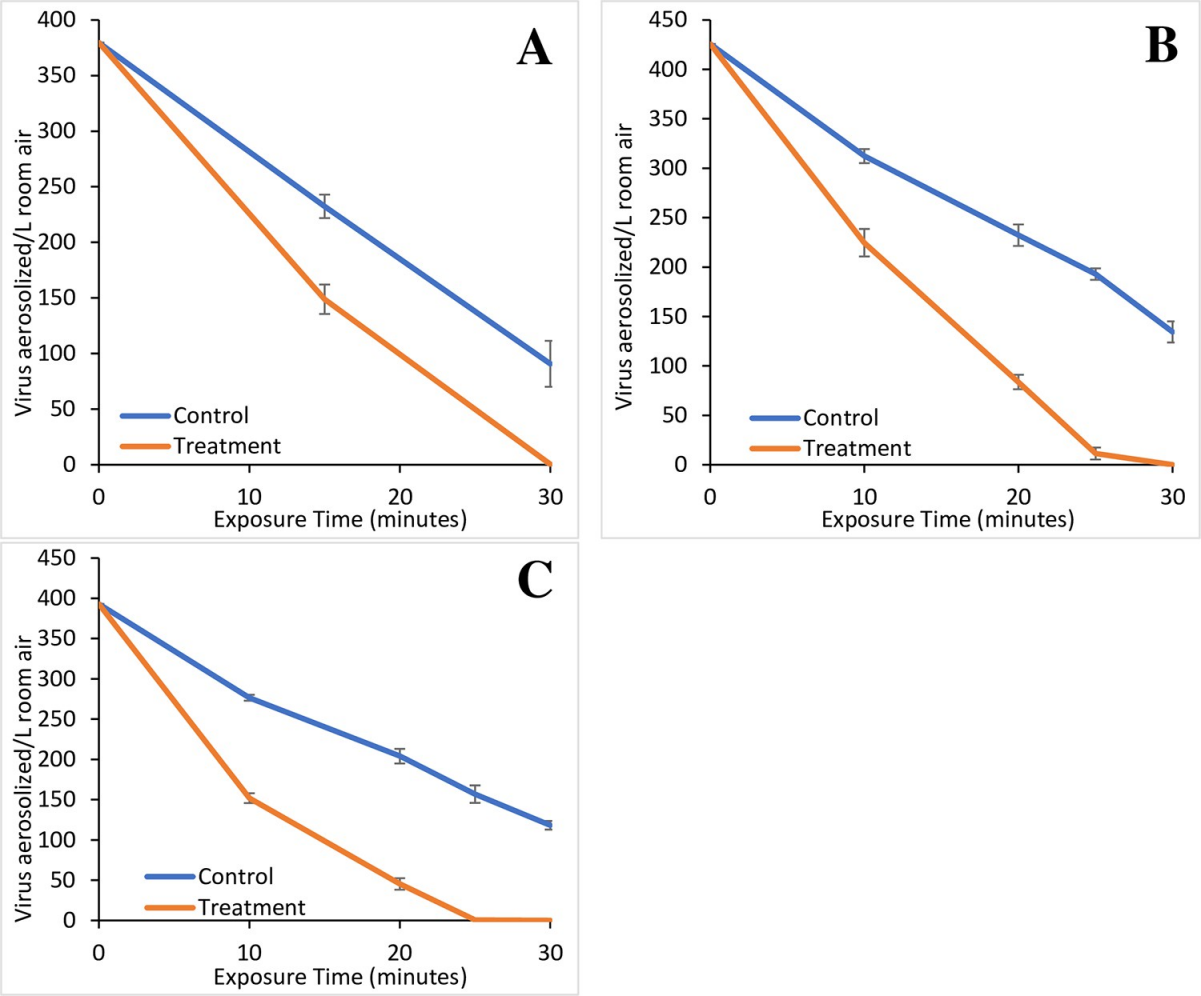
Loss of SARS viruses with ion treatment. The average loss over time of SARS-CoV-2 in the absence (control) and presence (treatment) of an operating GPS-FC48-AC at 4,900 negative ions/cm^3^(A), SARS-CoV-2 in the absence (control) and presence (treatment) of an operating GPS-FC48-AC at 12,000 negative ions/cm^3^(B), SARS-CoV-2 in the absence (control) and presence (treatment) of an operating GPS-FC48-AC at 18,000 negative ions/cm^3^(C). Plots are the average of triplicates for the control and treatment trials. Error bars represent the standard deviation of the replicates at each time interval.

Overall, the control trial replicates were similar in terms of TCID50/mL and for the viable virus/L of room air as shown for individual trials (Figs 4 and 5, S1 and S2 Figs in S1 File). The average values with standard deviation are shown in Figs 6 and 7. A Pearson correlation revealed that among replicate control trials, correlations were 0.983–0.999 with p values of 0.000–0.003 (S6 Table in S1 File). Further, correlations between all virus control trials showed correlations of 0.714–0.983 with p values of 0.000–0.009 (S6 Table in S1 File). These correlations highlight the stability of the test chambers because even with different pathogens and starting concentrations for a given virus, the natural decay in the control trials is highly correlated, which reflects chamber stability.

In general, the average Gross Virus Reduction Rate (GVRR) ranged from 20.6–25.9% after 30 minutes and 41.1–67.5% after 60 minutes (Table 1) for Influenza A and B, RSV, and SARS-CoV-2 Delta strain. Interestingly, there was greater natural loss of SARS-CoV-2 Alpha at 68.4–76.1% GRR after 30 minutes. Reasons for this are unclear since testing room conditions were the same at all times.

### Testing trials

The NPBI technology was assessed for its ability to effectively remove a variety of relevant viruses. For the test trials, as in the control trials, the 30 mL of virus preparation was divided into three 10 mL aliquots for the triplicate trials to be as similar as possible. In all cases, the operating device resulted in a substantial increase in loss of virus infectivity at 30 and 60 minutes (Table 1, S6 Table in S1 File and Figs 6 and 7, S3 and S4 Figs in S1 File). At 30 minutes, the Percent improvement (PI) ranged from 106.0–157.4% and 48.0–143.2% at 60 minutes for all but the SARS-CoV-2 Alpha strain (Table 1). The PI values using the TCID50/mL data were similar (S8 Table in S1 File). The PI was lower for the SARS-CoV-2 Alpha strain at 31.3–46.1% at 30 minutes even though this virus infectivity was reduced to a lower level than any other tested herein. This is primarily due to the considerably higher natural loss of virus activity in the controls (Table 1). Again, reasons for the higher natural loss of SARS-CoV-2 are unknown.

#### Influenza A

Influenza A was used to test the device for 60 minutes (Fig 6A, S3A Fig in S1 File). The NPBI technology reduced aerosolized Type A Influenza with 22,830 negative ions/cm^3^ and 12,415 particles/L (3.15x10^7^ TCID50/mL; Fig 6A, S3A Fig in S1 File and Tables 1 and 2, S7 and S8 Tables in S1 File). This viral load was reduced to 8,934±268 particles/L (2.27x10^7^±6.81x10^5^ TCID50/mL) at 15 minutes, 5,347±298 particles/L (1.36x10^7^±7.57x10^5^ TCID50/mL) at 30 minutes, 2,646±172 particles/L (6.71x10^6^±4.36x10^5^ TCID50/mL) after 45 minutes, and 17±8 particles/L (4.42x10^4^±1.94x10^3^ TCID50/mL) by the trial end at 60 minutes (Fig 6A, S3A Fig in S1 File and Table 2, S8 Table in S1 File). This represented an average reduction in recoverable Influenza A from the room air of 28.0±2.2% at 15 minutes, 56.9±2.4% at 30 minutes, 78.7±1.4% at 45 minutes, and 99.9±0.1% after 60 minutes of negative ion exposure (Table 3). This is a substantial increase compared to the control where only 11.9±2.5% of Influenza A was inactivated at 15 minutes, 22.1±1.9% at 30 minutes, 29.8±1.1% at 45 minutes and 41.1±6.0% was inactivated after 60 minutes (Table 3). This represented a 157.4% PI in viral removal after 30 minutes compared to the control trial, and a 143.2% PI after 60 minutes (Table 1). There were significant differences amongst all tests (P<0.01), except for the 30-minute and 60-minute tests in the amount of virus inactivated (S9 Table in S1 File Groupings, S10 Table in S1 File).

**Table 2:** Comparison of the average active virus concentration in control and test trials.

Virus/L					
		Control		Test	
Virus	Time (min)	Virus Particles/L	St Dev	Virus Particles/L	St Dev
Influenza A	0	3,476	-	3,476	-
Titer: 3.15E+07 TCID50/mL	15	3,064	89	2,502	75
	30	2,708	67	1,497	84
	45	2,439	40	741	48
	60	2,049	208	5	2
					
Influenza B	0	404	-	404	-
Titer: 3.66E+06 TCID50/mL	15	361	9	294	7
	30	321	4	208	12
	45	266	4	111	5
	60	209	3	36	13
					
RSV	0	458	-	458	-
Titer: 4.15E+06 TCID50/mL	15	390	9	337	12
	30	342	15	219	17
	45	278	24	124	13
	60	223	17	7	4
					
SARS-CoV-2 5K	0	106	-	106	-
Titer: 9.63E+05 TCID50/mL	15	65	4	42	5
	30	25	8	0	0
					
SARS-CoV-2 12K	0	119	-	119	-
Titer: 1.08E+06 TCID50/mL	10	87	3	63	6
	20	65	4	23	3
	25	54	2	3	2
	30	38	4	0	-
					
SARS-CoV-2 18K	0	110	-	110	-
Titer: 1.08E+06 TCID50/mL	10	77	1	43	2
	20	57	3	13	2
	25	44	3	0	0
	30	33	1	0	0
					
SARS-CoV-2 Delta	0	2,726	-	2,726	-
Titer: 2.47E+07 TCID50/mL	15	2,325	50	1,950	32
	30	2,020	83	1,095	75
	45	1,538	106	441	97
	60	885	91	0	0

**Table 3:** Percent viral loss over time for all control and trial experiments.

	Control	Test	Control	Test	Control	Test	Control	Test	Control	Test	Control	Test	Control	Test
Virus	10	10	15	15	20	20	25	25	30	30	45	45	60	60
Influenza A			11.9±2.5	28.0±2.2					22.1±1.9	56.9±2.4	29.8±1.1	78.7±1.4	41.1±6.0	99.9±0.1
Influenza B			10.7±2.2	27.3±1.8					20.6±1.1	48.5±3.0	34.2±1.1	72.5±1.1	48.4±0.7	91.1±3.1
RSV			14.8±1.9	26.3±2.7					25.3±3.2	52.1±3.8	39.2±5.3	73.0±2.9	51.2±3.7	98.6±0.9
SARS-CoV-2 5K			38.8±4.0	60.8±5.0					76.1±7.8	99.9±0.1				
SARS-CoV-2 12K	26.6±2.4	47.2±4.7			45.4±3.7	80.3±2.5	54.7±2.0	97.3±2.0	68.4±3.6	100.0±0.0				
SARS-CoV-2 18K	29.6±0.9	61.4±1.6			48.1±2.3	88.5±1.8	60.1±1.4	100.0±0.0	69.9±1.4	100.0±0.0				
SARS-CoV-2 Delta			14.7±1.8	28.5±1.2					25.9±3.1	59.8±2.7	43.6±3.9	83.8±3.6	67.5±3.3	100.0±0.0

#### Influenza B

Throughout the 60-minute trial, the results showed faster virus inactivation than observed with natural loss (Fig 6B, S3B Fig in S1 File and Tables 1–3, S7 and S8 Tables in S1 File) using 12,900 positive and 22,850 negative ions /cm^3^ with 1,443 particles/L (3.66 x 10^6^ TCID50/mL; Table 1, S6 Table in S1 File and Fig 6B, S3B Fig in S1 File). Influenza B was reduced to 1,048±26 particles/L (2.66x10^6^±6.56x10^4^ TCID50/mL) after 15 minutes, 744±43 particles/L (1.89x10^6^±1.10x10^5^ TCID50/mL) after 30 minutes, 396±16 particles/L (1.01x10^6^±4.11x10^4^ TCID50/mL) after 45 minutes, and 128±45 particles/L (3.25x10^5^±1.15x10^5^ TCID50/mL) after 60 minutes (Fig 6B, S3B Fig in S1 File and Table 2, S8 Table in S1 File). These values correlated to a percent virus inactivated in the test and control trials, respectively, of 27.3±1.8% versus 10.7±2.2% at 15 minutes, 48.5±3.0% versus 20.6±1.1% at 30 minutes, 72.5±1.1% versus 34.2±1.1% at 45 minutes and 91.1±3.1% versus 48.4±0.7% at the 60-minute end (Table 3). In terms of overall virus inactivated, the results showed a PI of 135.4% at 30 minutes and 88.4% at 60 minutes (Table 1). As with Influenza A there were significant differences amongst all tests (P<0.01), except for the 30-minute and 60-minute tests in the amount of virus inactivated (S9 Table in S1 File Groupings, S10 Table in S1 File).

#### Human Respiratory Syncytial Virus (RSV)

The device was operated at 9,600 positive and 23,600 negative ions /cm^3^ using a starting concentration of 1,636 particles/L (4.15x10^6^ TCID50/mL; Table 1, S6 Table in S1 File). The results showed that ion exposure consistently reduced aerosolized RSV (Fig 6C, S3C Fig in S1 File and Tables 1–3, S7 and S8 Tables in S1 File). The RSV concentration was reduced to 1,205±43 particles/L (3.06x10^6^±1.10x10^5^ TCID50/mL after 15 minutes, to 783±62 particles/L (1.99x10^6^±1.58x10^5^ TCID50/mL) after 30 minutes, to 441±48 particles/L (1.12x10^6^±1.21x10^5^ TCID50/mL) after 45 minutes, and to 23±14 particles/L (5.96x10^4^±3.64x10^4^ TCID50/mL) after 60 minutes (Fig 6C, S3C Fig in S1 File and Tables 1–3, S7 Table in S1 File). The percent of aerosolized virus inactivated, in the test and control trials, respectively, was 26.3±2.7% versus 14.8±1.9% at 15 minutes, 52.1±3.8% versus 25.3±3.2% at 30 minutes, 73.0±2.9% versus 39.2±5.3% at 45 minutes and 98.6±0.9% versus 51.2±3.7% at 60 minutes (Table 3). In a similar fashion to Influenza A and B, the exposure to ions increased RSV inactivated from the room air yielding a PI of 106.0% at 30 minutes and 92.3% at 60 minutes from the control trials (Table 1). RSV results were also significant and showed similar losses of RSV at the 15-minute test and 30-minute control as well as at the 30-minute test and 60-minute control at p<0.01 (S9 Table in S1 File Groupings, S10 Table in S1 File) accentuating the increased viral loss with ionization.

#### SARS-CoV-2 Delta strain

The Delta strain closely resembled the other viruses tested with respect to the natural loss observed in the control trials (Tables 1–3, S7 and S8 Tables in S1 File and Fig 6, S3 Fig in S1 File). The GPS-FC48-AC was operated at 15,790 positive ions/cm^3^ and 24,070 negative ions/cm^3^. Within 15 minutes the virus was reduced to 6,963±114 particles/L (1.77x10^7^±2.89x10^5^ TCID50/mL) from the initial 9,735 particles/L (2.47x10^7^ TCID50/mL), representing a 28.5±1.2% loss as compared to the 14.7±1.8% loss in the control (Tables 2 and 3). After 30 minutes the virus count was reduced to 3,910±267 particles/L (9.92x10^6^±6.78x10^5^ TCID50/mL), to 1,575±348 particles/L (4.00x10^6^±8.83x10^5^ TCID50/mL) after 45 minutes and finally to 1±0 (1.57x10^3^±8.09x10^2^ TCID50/mL) at 60 minutes (Table 2, S8 Table in S1 File). This yielded a 59.8±2.7%, 83.8±3.6%, and 99.9±0.1% virus elimination, respectively (Fig 6D, S3D Fig in S1 File and Table 3), correlating to a PI of 130.9% after 30 minutes and 48.0% after 60 minutes over the observed natural loss (Table 1). Statistical significance mirrored the RSV results in that similarity was found between the 15-minute test and 30-minute control as well as at the 30-minute test and 60-minute control at p<0.01 (S9 Table in S1 File Groupings and S10 Table in S1 File).

#### SARS-CoV-2 Alpha strain

The GPS-FC48-AC was first operated at 4,900 negative ions/cm^3^, then at 12,000 ions/cm^3^, and then 18,000 ions/cm^3^ over 30 minutes. At 4,900 ions/cm^3^, after 15 minutes viable Alpha strain was reduced to 149±19 particles/L (3.78x10^5^±4.80x10^4^ TCID50/mL) from a starting concentration of 380 particles/L (9.63x10^5^ TCID50/mL; Fig 7A, S4A Fig in S1 File and Tables 1 and 2, S7 and S8 Tables in S1 File), a 60.8+5.0% loss of viable virus (Table 3). By 30 minutes, 0±0 particles/L (1.13x10^3^±9.02x10^2^ TCID50/mL) remained (Table 2, S8 Table in S1 File) and represented a 99.9±0.1% removal of the virus from the indoor air space (Table 3). At 15 minutes 60.8±5.0% of the virus was removed compared to 38.8±4.0% in the control and this continued at 30 minutes with 99.9±0.1% of the virus removed by the device while only 76.1±7.8% was removed by natural loss (Table 3). This represented a PI of 31.3% at this low ion setting (Table 1, S6 Table in S1 File). While the trial was initially run for 60 minutes as with other trials in this study, the 45- and 60-minute data is not shown as it fell below the level of detection. Here each of the time points for the controls and tests were significant from each other (p<0.01, S9 Table in S1 File Groupings, S10 Table in S1 File), highlighting the effect of ionization but also likely because there were only two time points.

Given the results at 4,900 ions/cm^3^, we limited the higher-powered trials to 30 minutes and took more frequent samples to better ascertain viable viral loss rates. With a negative ion level of 12,000 ions/cm^3^, samples were taken at 0, 10, 20, 25 and 30 minutes (Fig 7B, S4B Fig in S1 File and Tables 2, 3 and S8 Table in S1 File). By 10 minutes the Alpha strain concentration was reduced to 225±20 particles/L (5.70x10^5^±5.05x10^4^ TCID50/mL) from the starting concentration of 426 particles/L (1.08x10^6^ TCID50/mL), a 47.2±4.7% loss as compared to 26.6±2.4% loss in the control (Tables 2 and 3). After 20 minutes, 84±10 particles/L (2.12x10^5^±2.65x10^4^ TCID50/mL) remained and corresponded to an 80.3±2.5% loss, nearly double the 45.4±3.7% in the control. At 25 minutes, only 11±9 particles/L (2.88x10^4^±2.19x10^4^ TCID50/mL) remained, representing a 97.3±2.0% clearance as compared to 54.7±2.0% in the control. Finally, at 30 minutes viral concentrations were below detection with a clearance of 99.9±0.1% as compared to the control of 68.4±3.6% (Tables 1–3, S7 and S8 Tables in S1 File), representing a PI of 46.1% (Table 1). Statistically, the viral loss at 10-minutes with ionization was similar to the 20- and 25-minute controls (p<0.01), continuing the significant trend shown with the other viruses tested herein (S9 Table in S1 File Groupings, S10 Table in S1 File).

Based upon the increase in the PI from 31.3%-46.1% with increasing the ion intensity from 4,900 ions/cm^3^ to 12,000 ions/cm^3^ (Table 1), we performed a third trial with 18,000 negative ions/cm^3^. The performance was similar to the 12,000 ions/cm^3^ trials (Fig 7C, S4C Fig in S1 File and Tables 1–3, S7 and S8 Tables in S1 File). Starting with 393 particles/L (9.97x10^5^ TCID50/mL), it was reduced to 152±6 particles/L (3.85x10^5^±1.56x10^4^ TCID50/mL) after 10 minutes, to 45±7 particles/L (1.15x10^5^±1.81x10^4^ TCID50/mL) at 20 minutes, to 1±0 particles/L (1.31x10^3^±1.17x10^2^ TCID50/mL) at 25 minutes and below detection at 30 minutes (Fig 7C, S4C Fig in S1 File and Table 2, S8 Table in S1 File). These results represent similar viable virus percent losses to the 12,000 ions/cm^3^ trials (Table 3) with a PIs of 43.0% at 18,000 ions/cm^3^ and 46.1% at 12,000 ions/cm^3^ (Table 1). Again, this increase was substantially smaller than the PI of 106.0–157.4% observed with the other virus trials. This is primarily due to the relatively quick natural Gross Reduction Rate in the controls of 68.4–76.1% by 30 minutes, as compared to the other trials where only 20.6–25.9% of the viable virus was lost in the controls at 30 minutes (Table 3). The significance observed at 18,000 ions/cm^3^ was similar to 12,000 ions/cm^3^ in that the only similarity was with the 10-minute test and the 25-minute control (p<0.01, S9 Table in S1 File Groupings and S10 Table in S1 File).

## Discussion

The goal of this study was to determine the effectiveness of ion exposure using the NPBI bipolar ion technology to reduce the infectivity of SARS-CoV-2 Alpha and Delta strains, Influenza A, Influenza B, and RSV. Influenza A and Influenza B are prevalent yearly [24, 25], as is the Human Respiratory Syncytial Virus (RSV) [26] with >3,000,000 US cases annually and serious infection risk to children >2 years old [27]. So then, in light of ongoing outbreaks with each virus [28–30] and particularly SARS-CoV-2 [31–37], it is crucial to determine how effective different methods can be on cleaning indoor air to provide clean and safe environments.

The effectiveness of the bipolar ionization treatment was determined by the ion to particle ratio. The carrier trial shown in Fig 3 served as a baseline for demonstrating ion suppression. The aerosolized virus particles introduced more ultrafine particles which eventually overwhelmed the available ions, resulting in ion suppression. So then an artificially high virus concentration in the high 6 Log to 10 Log, which is commonly used in laboratory testing [38], causes significant ion suppression and severely limits the ion rebound effect. Hence, these artificially high virus concentrations bias the net pathogen reduction, suggesting device underperformance. To determine the true efficacy, realistic concentrations of virus are required.

Virus concentrations used here ranged from ~100–3,500 virus particles/L, far lower than other studies. The typical human breath or sneeze is ~500mL [39]. With SARS-CoV-2, concentrations can be as high as 1,800–3,400 virus particles/m^3^ in infected hospital rooms [34]. This translates to 2–4 virus particles/L, lower than used in this study. However, an infected person can aerosolize ~200–7,000 virus particles from breathing and casual conversation within a 2-meter area [40], which is consistent with the virus concentrations used in our study.

Regarding the accentuated SARS-CoV-2 Alpha natural decay rate, a literature review did not provide evidence of a size difference between the Alpha and the Delta particles ranging from 0.075–0.16 microns [41–43]. While there are 23 known genetic mutations between the two strains, much of the focus has been on the infectious spike protein [42–46]. One explanation that may resolve part of the Alpha strain rapid natural loss is airborne contaminants. Part of the SARS-CoV-2 Alpha contagiousness is linked to the virus attaching to particulate matter, NO_2_, CO, SO_2_ and ground-level O_3_ [36]. Particulate matter (<10 microns) concentrations were highly correlated with increased in Covid19 infections in northern Italy [47]. Since our tests were in a sealed clean room, there should have been no particles for the virus attachment. However, this still does not completely explain why the viable Alpha strain numbers decreased more quickly than the others. When aerosolizing pathogens, some variables cannot be fully accounted for, namely pathogen placement, collection volume, collection points, drop rate, surface saturation, viral destruction on collection, viral destruction on aerosolization, and possibly others. Every effort was made to address these constraints in the experimental design and is reflected in the results.

## Conclusions

The object of large space laboratory virus studies is to obtain efficacy data that is relevant to real world environments. Most published device chamber studies that claim to reduce airborne pathogens used unrealistically high viral concentrations, which may result in an under-performance bias, and may be especially true for bipolar ionization devices that function by instantaneous interaction with particles in the enclosed space. This practice not only biases the study but presents the end consumer with inaccurate and perhaps misleading information regarding the effectiveness of a given technology. Such pitfalls serve to stifle the development of innovative technologies that may greatly reduce infection rates and save lives. Hence to accurately assess the true efficacy in a relevant manner, these studies must incorporate real-world levels of airborne viruses and/or non-biological particles.

The results of this study lead to four succinct conclusions in that first, bipolar ionization is effective for reducing infectious airborne viruses in large indoor spaces since, secondly, all ion levels tested significantly reduced virus infectivity. Third, the real-world virus concentrations used resulted in rapid inactivation of respiratory virus as compared to artificially high laboratory concentrations. Finally, the large space laboratory testing results provide solid evidence of the benefit of NPBI when used to reduce respiratory virus infectivity in occupied spaces.

## Supporting information

S1 File (DOCX)
