# Supplementary material for: Bipolar ionization rapidly inactivates real-world, airborne concentrations of infective respiratory viruses
Source: PLoS One. 2023 Nov 22;18(11):e0293504. doi: 10.1371/journal.pone.0293504 (PMC10664916; doi:10.1371/journal.pone.0293504)
Supplement: S1 File — (DOCX) [file pone.0293504.s001.docx]

**Supplemental Information for Bipolar Ionization Rapidly Inactivates Real-World, Airborne Concentrations of Infective Respiratory Viruses**

**C**

**D**

**A**

**B**

**Figure S1: Triplicate control runs of viruses.**

Control runs to determine the TCID50 natural loss of (A) Influenza A, (B) Influenza B, (C) RSV, and (D) the SARS-CoV-2 Delta variant, demonstrating the similarity and repeatability of the system used in this study. Vertical axis is expressed in millions.

**A**

**B**

**C**

**Figure S2: Triplicate control runs for SARS viruses.**

Control runs to determine the TCID50 natural loss of (A) SARS-CoV-2 to compare with the GPS-FC48-AC operating at 4,900 negative ions/cm^3^, (B) SARS-CoV-2 to compare with the GPS-FC48-AC operating at 12,000 negative ions/cm^3^, and (C) SARS-CoV-2 to compare with the GPS-FC48-AC operating at 18,000 negative ions/cm^3^, demonstrating the similarity and repeatability of the system used in this study. Vertical axis is expressed in millions.

**Figure S3: Comparison of TCID50 loss in controls and treatments.**


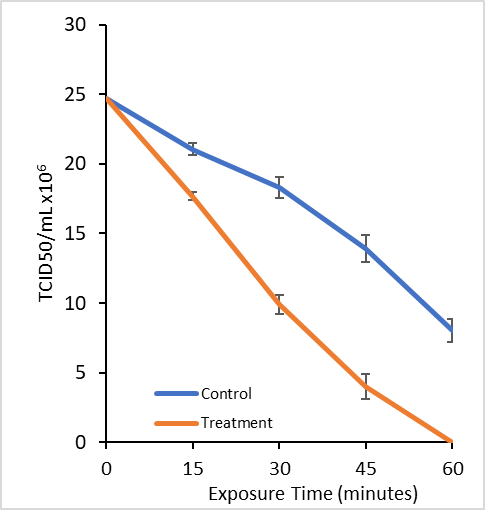


**D**

**A**

**B**

**C**

The average TCID50 loss over time of (A) Influenza A in the absence (control) and presence (treatment) of an operating GPS-FC48-AC, (B) Influenza B in the absence (control) and presence (treatment) of an operating GPS-FC48-AC, (C) RSV in the absence (control) and presence (treatment) of an operating GPS-FC48-AC. (D) SARS-CoV-2 Delta variant in the absence (control) and presence (treatment) of an operating GPS-FC48-AC. Plots are the average of triplicates for the control and treatments. Vertical axis is expressed in millions. Error bars represent the standard deviation of the replicates at each time interval.

**C**

**B**

**A**

**Figure S4:** **Comparison of TCID50 loss in controls and treatments at different ion strengths.**

The average TCID50 loss over time of (A) SARS-CoV-2 in the absence (control) and presence (treatment) of an operating GPS-FC48-AC at 4,900 negative ions/cm^3^, (B) SARS-CoV-2 in the absence (control) and presence (treatment) of an operating GPS-FC48-AC at 12,000 negative ions/cm^3^, (C) SARS-CoV-2 in the absence (control) and presence (treatment) of an operating GPS-FC48-AC at 18,000 negative ions/cm^3^. Plots are the average of triplicates for the control and treatment trials. Vertical axis is expressed in millions. Error bars represent the standard deviation of the replicates at each time interval.

**Table S1: Preparation and Testing of Influenza A**

| Viral Stock: Influenza A Virus (BEI NR-31132)  **TEST** | **SPECIFICATIONS** | **RESULTS** |
| --- | --- | --- |
| **Identification by Infectivity Using Embryonated Chicken Eggs**  Hemagglutination activity using allantoic fluid from infected eggs and 0.5% chicken red blood cells | Positive | Positive |
| **Sequencing of Hemagglutinin, Matrix, and Neuraminidase Coding Regions**  Hemagglutinin (689 nucleotides) | Consistent with  A /Wisconsin/67/2009 (H3N2) | 99% identity with  A/Wisconsin/67/2009 (H3N2) GenBank: CY163648 |
| Matrix (911 nucleotides) | Consistent with  A /Wisconsin/67/2009 (H3N2) | 99% identity with  A/Wisconsin/67/2009 (H3N2) GenBank: CY163648 |
| Neuraminidase (445 nucleotides) | Consistent with  A /Wisconsin/67/2009 (H3N2) | 99% identity with  A/Wisconsin/67/2009 (H3N2) GenBank: CY163648 |
| **Titer by CEID50 in Embryonated Chicken Eggs** | Report Results | 2.8 X 108 CEID50 per mL |
| **Sterility (21-Day Incubation)**  Harpos HTYE Broth, aerobic | No Growth | No Growth |
| Trypticase Soy Broth, aerobic | No Growth | No Growth |
| Sabourad Broth, aerobic | No Growth | No Growth |
| Sheep Blood Agar, aerobic | No Growth | No Growth |
| Sheep Blood Agar, anaerobic | No Growth | No Growth |
| Thioglycollate Broth, anaerobic | No Growth | No Growth |
| DMEM with 10% FBS | No Growth | No Growth |
| **Mycoplasma Contamination**  Agar and Broth Culture | None Detected | None Detected |
| DNA Detection by PCR of extracted test article nucleic acid | None Detected | None Detected |

| Viral Stock: Influenza B Virus (BEI NR-48660)  **TEST** | **SPECIFICATIONS** | **RESULTS** |
| --- | --- | --- |
| **Identification by Infectivity in MDCK Cells** | Cell rounding and detachment | Cell rounding and detachment |
| **Sequencing of Neuraminidase Coding Regions**  (~900 nucleotides) | > 98% identity with B/New York/1055/2003  GenBank: CY174331.1 | 100% identity with B/New York/1055/2003  GenBank: CY174331.1 |
| **Titer by TCID50 Assay in MDCK Cells by Cytopathic Effect** | Report Results | 5 X 106 TCID50 per mL |
| **Sterility (21-Day Incubation)**  Harpos HTYE Broth, aerobic | No Growth | No Growth |
| Trypticase Soy Broth, aerobic | No Growth | No Growth |
| Sabourad Broth, aerobic | No Growth | No Growth |
| Sheep Blood Agar, aerobic | No Growth | No Growth |
| Sheep Blood Agar, anaerobic | No Growth | No Growth |
| Thioglycollate Broth, anaerobic | No Growth | No Growth |
| DMEM with 10% FBS | No Growth | No Growth |
| **Mycoplasma Contamination**  Agar and Broth Culture | None Detected | None Detected |
| DNA Detection by PCR of extracted test article nucleic acid | None Detected | None Detected |

**Table S2: Preparation and Testing of Influenza B**

*The viral titer listed in the Certificate of Analysis represents the titer provided by BEI Resources.

**Table S3: Preparation and Testing of Human Respiratory Syncytial Virus**

| Viral Stock: Human Respiratory Syncytial Virus (NR-28525, Lot #: 60109225) **TEST** | **SPECIFICATIONS** | **RESULTS** |
| --- | --- | --- |
| **Identification by Infectivity in HE-p2 Cells** | Cell rounding and sloughing | Cell rounding and sloughing |
| **Sequencing of Species-Specific Region** (822 nucleotides) | Consistent with human respiratory syncytial virus, A2001/2-20 | Consistent with human respiratory syncytial virus, A2001/2-20  GenBank: JX069798 |
| **Titer by TCID50 in Hep-2 Cells** | Report Results | 2.8 X 108 TCID50 per mL |
| **Sterility (21-Day Incubation)**  Harpos HTYE Broth, aerobic | No Growth | No Growth |
| Trypticase Soy Broth, aerobic | No Growth | No Growth |
| Sabourad Broth, aerobic | No Growth | No Growth |
| Sheep Blood Agar, aerobic | No Growth | No Growth |
| Sheep Blood Agar, anaerobic | No Growth | No Growth |
| Thioglycollate Broth, anaerobic | No Growth | No Growth |
| DMEM with 10% FBS | No Growth | No Growth |
| **Mycoplasma Contamination**  Agar and Broth Culture | None Detected | None Detected |
| DNA Detection by PCR of extracted test article nucleic acid | None Detected | None Detected |

*The viral titer listed in the Certificate of Analysis represents the titer provided by BEI Resources.

**Table S4: Preparation and Testing of SARS-CoV-2 Alpha Strain**

| Viral Stock: SARS-CoV-2 USA-CA1/2020 (BEI NR-52382)  **TEST** | **SPECIFICATIONS** | **RESULTS** |
| --- | --- | --- |
| **Identification by Infectivity in Vero 6 Cells** | Cell Rounding and Detachment | Cell Rounding and Detachment |
| **Next-Generation Sequencing (NGS) of the complete genome using Illumina® iSeq™ 100 Platform** | ≥ 98% identity with SARS-CoV 2, isolate USA-CA1/2020  GenBank: MN994467.1 | 99.9% identity with SARS-CoV 2, isolate USA-CA1/2020  GenBank: MN994467.1 |
| Approx. 940 Nucleotides | ≥ 98% identity with SARS-CoV 2, strain FDAARGOS_983 isolate USA-CA1/2020  GenBank: MT246667.1 | 100% identity with SARS-CoV 2, strain FDAARGOS_983 isolate USA-CA1/2020  GenBank: MT246667.1 |
| **Titer by TCID50 in Vero E6 Cells by Cytopathic Effect** | Report Results | 2.8 X 105 TCID50 per mL in 5 days at 37°C and 5% CO2 |
| **Sterility (21-Day Incubation)**  Harpos HTYE Broth, aerobic | No Growth | No Growth |
| Trypticase Soy Broth, aerobic | No Growth | No Growth |
| Sabourad Broth, aerobic | No Growth | No Growth |
| Sheep Blood Agar, aerobic | No Growth | No Growth |
| Sheep Blood Agar, anaerobic | No Growth | No Growth |
| Thioglycollate Broth, anaerobic | No Growth | No Growth |
| DMEM with 10% FBS | No Growth | No Growth |
| **Mycoplasma Contamination**  Agar and Broth Culture | None Detected | None Detected |
| DNA Detection by PCR of extracted test article nucleic acid | None Detected | None Detected |

*The viral titer listed in the Certificate of Analysis is representative of the titer provided by BEI Resources. These viruses are grown on VeroE6 cells either in-house or at a partner lab to the concentrations listed within the experiment design.

**Table S5: Preparation and Testing of SARS-CoV-2 Delta Strain**

| Viral Stock: SARS-CoV-2 Delta Variant (BEI NR-55611)  **TEST** | **SPECIFICATIONS** | **RESULTS** |
| --- | --- | --- |
| **Identification by Infectivity in Calu-3 Cells** | Cell rounding and detachment | Cell rounding and detachment |
| **Next-Generation Sequencing (NGS) of Complete Genome Using Illumina® iSeq™ 100 Platform** | > 98% identity with SARS-CoV-2, hCoV-19/USA/PHC658/2021 depositor sequence | 99.99% identity with SARS-CoV-2, hCoV-19/USA/PHC658/2021 depositor sequence |
| **Titer by TCID50 Assay in Calu-3 Cells by Cytopathic Effect** | Report Results | 6.5 X 105 TCID50 per mL |
| **Sterility (21-Day Incubation)**  Harpo's HTYE Broth, aerobic | No Growth | No Growth |
| Trypticase Soy Broth, aerobic | No Growth | No Growth |
| Sabourad Broth, aerobic | No Growth | No Growth |
| Sheep Blood Agar, aerobic | No Growth | No Growth |
| Sheep Blood Agar, anaerobic | No Growth | No Growth |
| Thioglycollate Broth, anaerobic | No Growth | No Growth |
| DMEM with 10% FBS | No Growth | No Growth |
| **Mycoplasma Contamination**  Agar and Broth Culture | None Detected | None Detected |
| DNA Detection by PCR of extracted test article nucleic acid | None Detected | None Detected |

*The viral titer listed in the Certificate of Analysis represents the titer provided by BEI Resources.

**Table S6: Pearson Correlations of Individual Virus and Between Virus Control Trials**

| **Pairwise Pearson Correlations for control replicates across viruses** | | | | | |
| --- | --- | --- | --- | --- | --- |
| Sample 1 | Sample 2 | N | Correlation | 95% CI for ρ | P-Value |
| Influenza B | Influenza A | 12 | 0.714 | (0.238, 0.914) | 0.009 |
| RSV | Influenza A | 12 | 0.903 | (0.684, 0.973) | 0 |
| SARS-Delta | Influenza A | 12 | 0.925 | (0.750, 0.979) | 0 |
| RSV | Influenza B | 12 | 0.719 | (0.247, 0.915) | 0.008 |
| SARS-Delta | Influenza B | 12 | 0.728 | (0.265, 0.918) | 0.007 |
| SARS-Delta | RSV | 12 | 0.983 | (0.940, 0.995) | 0 |
|  |  |  |  |  |  |
| **Pairwise Pearson Correlations for influenza A replicate controls** | | | | | |
| Sample 1 | Sample 2 | N | Correlation | 95% CI for ρ | P-Value |
| Replicate 2 | Replicate 1 | 5 | 0.997 | (0.947, 1.000) | 0 |
| Replicate 3 | Replicate 1 | 5 | 0.986 | (0.802, 0.999) | 0.002 |
| Replicate 3 | Replicate 2 | 5 | 0.983 | (0.754, 0.999) | 0.003 |
|  |  |  |  |  |  |
| **Pairwise Pearson Correlations for influenza B replicate controls** | | | | | |
| Sample 1 | Sample 2 | N | Correlation | 95% CI for ρ | P-Value |
| Replicate 2 | Replicate 1 | 5 | 0.996 | (0.931, 1.000) | 0 |
| Replicate 3 | Replicate 1 | 5 | 0.999 | (0.986, 1.000) | 0 |
| Replicate 3 | Replicate 2 | 5 | 0.997 | (0.958, 1.000) | 0 |
|  |  |  |  |  |  |
| **Pairwise Pearson Correlations for SARS-Delta** **replicate controls** | | | | | |
| Sample 1 | Sample 2 | N | Correlation | 95% CI for ρ | P-Value |
| Replicate 2 | Replicate 1 | 5 | 0.996 | (0.931, 1.000) | 0 |
| Replicate 3 | Replicate 1 | 5 | 0.999 | (0.986, 1.000) | 0 |
| Replicate 3 | Replicate 2 | 5 | 0.997 | (0.958, 1.000) | 0 |
|  |  |  |  |  |  |
| **Pairwise Pearson Correlations for RSV replicate controls** | | | | | |
| Sample 1 | Sample 2 | N | Correlation | 95% CI for ρ | P-Value |
| Replicate 2 | Replicate 1 | 5 | 0.996 | (0.931, 1.000) | 0 |
| Replicate 3 | Replicate 1 | 5 | 0.999 | (0.986, 1.000) | 0 |
| Replicate 3 | Replicate 2 | 5 | 0.997 | (0.958, 1.000) | 0 |
|  |  |  |  |  |  |

Table S7: Ions Levels Used in all Trials with Average TCID50 Viral Loss over Time

Table S8: Comparison of Average TCID50 Viral Load in Control and Test Trials

Table S9: Statistical Analyses of the Effect of Ionization on Viral Loss over Time

| **Influenza A** |  |  |  |  |  |  |  |  |  |
| --- | --- | --- | --- | --- | --- | --- | --- | --- | --- |
| *Analysis of Variance* |  |  |  |  |  |  |  |  |  |
| **Source** | **DF** | **Adj SS** | **Adj MS** | **F-Value** | **P-Value** |  |  |  |  |
| Exposure Time | 3 | 128,296,225 | 42,765,408 | 371 | 0 |  |  |  |  |
| NPBI | 1 | 145,493,745 | 145,493,745 | 1,262 | 0 |  |  |  |  |
| Exposure Time*NPBI | 3 | 23,700,923 | 7,900,308 | 69 | 0 |  |  |  |  |
| Error | 16 | 1,844,635 | 115,290 |  |  |  |  |  |  |
| Total | 23 | 299,335,528 |  |  |  |  |  |  |  |
|  |  |  |  |  |  |  |  |  |  |
| *Grouping Information Using the Tukey Method and 95% Confidence* | | | | |  |  |  |  |  |
| **Exposure Time*NPBI** | **N** | **Mean** | **Grouping** | | | | | | |
| 15 Control | 3 | 10,944 | A |  |  |  |  |  |  |
| 30 Control | 3 | 9,670 |  | B |  |  |  |  |  |
| 15 Test | 3 | 8,934 |  | B |  |  |  |  |  |
| 45 Control | 3 | 8,710 |  | B |  |  |  |  |  |
| 60 Control | 3 | 7,318 |  |  | C |  |  |  |  |
| 30 Test | 3 | 5,347 |  |  |  | D |  |  |  |
| 45 Test | 3 | 2,646 |  |  |  |  | E |  |  |
| 60 Test | 3 | 17 |  |  |  |  |  | F |  |
| ***Means that do not share a letter are significantly different.* | | | | | | | | | |
| **Influenza B** |  |  |  |  |  |  |  |  |  |
| *Analysis of Variance* |  |  |  |  |  |  |  |  |  |
| **Source** | **DF** | **Adj SS** | **Adj MS** | **F-Value** | **P-Value** |  |  |  |  |
| Exposure Time | 3 | 1,830,174 | 610,058 | 756 | 0 |  |  |  |  |
| NPBI | 1 | 1,230,517 | 1,230,517 | 1,525 | 0 |  |  |  |  |
| Exposure Time*NPBI | 3 | 126,666 | 42,222 | 52 | 0 |  |  |  |  |
| Error | 16 | 12,908 | 807 |  |  |  |  |  |  |
| Total | 23 | 3,200,265 |  |  |  |  |  |  |  |
|  |  |  |  |  |  |  |  |  |  |
| *Grouping Information Using the Tukey Method and 95% Confidence* | | | | |  |  |  |  |  |
| **Exposure Time*NPBI** | **N** | **Mean** | **Grouping** | | | | | | |
| 15 Control | 3 | 1,289 | A |  |  |  |  |  |  |
| 30 Control | 3 | 1,146 |  | B |  |  |  |  |  |
| 15 Test | 3 | 1,048 |  |  | C |  |  |  |  |
| 45 Control | 3 | 949 |  |  |  | D |  |  |  |
| 60 Control | 3 | 745 |  |  |  |  | E |  |  |
| 30 Test | 3 | 744 |  |  |  |  | E |  |  |
| 45 Test | 3 | 396 |  |  |  |  |  | F |  |
| 60 Test | 3 | 128 |  |  |  |  |  |  | G |
| ***Means that do not share a letter are significantly different.* | | | | | | | | | |
| **RSV** |  |  |  |  |  |  |  |  |  |
| *Analysis of Variance* |  |  |  |  |  |  |  |  |  |
| **Source** | **DF** | **Adj SS** | **Adj MS** | **F-Value** | **P-Value** |  |  |  |  |
| Exposure Time | 3 | 2,613,110 | 871,037 | 300 | 0 |  |  |  |  |
| NPBI | 1 | 1,433,386 | 1,433,386 | 494 | 0 |  |  |  |  |
| Exposure Time*NPBI | 3 | 266,607 | 88,869 | 31 | 0 |  |  |  |  |
| Error | 16 | 46,412 | 2,901 |  |  |  |  |  |  |
| Total | 23 | 4,359,515 |  |  |  |  |  |  |  |
|  |  |  |  |  |  |  |  |  |  |
| *Grouping Information Using the Tukey Method and 95% Confidence* | | | | |  |  |  |  |  |
| **Exposure Time*NPBI** | **N** | **Mean** | **Grouping** | | | | | |  |
| 15 Control | 3 | 1,394 | A |  |  |  |  |  |  |
| 30 Control | 3 | 1,222 |  | B |  |  |  |  |  |
| 15 Test | 3 | 1,205 |  | B |  |  |  |  |  |
| 45 Control | 3 | 995 |  |  | C |  |  |  |  |
| 60 Control | 3 | 797 |  |  |  | D |  |  |  |
| 30 Test | 3 | 783 |  |  |  | D |  |  |  |
| 45 Test | 3 | 441 |  |  |  |  | E |  |  |
| 60 Test | 3 | 23 |  |  |  |  |  | F |  |
| ***Means that do not share a letter are significantly different.* | | | | | | | | | |
| **SARS-CoV-2 Delta Variant** | |  |  |  |  |  |  |  |  |
| *Analysis of Variance* |  |  |  |  |  |  |  |  |  |
| **Source** | **DF** | **Adj SS** | **Adj MS** | **F-Value** | **P-Value** |  |  |  |  |
| Exposure Time | 3 | 122,272,723 | 40,757,574 | 567 | 0 |  |  |  |  |
| NPBI | 1 | 51,495,015 | 51,495,015 | 717 | 0 |  |  |  |  |
| Exposure Time*NPBI | 3 | 5,538,570 | 1,846,190 | 26 | 0 |  |  |  |  |
| Error | 16 | 1,149,837 | 71,865 |  |  |  |  |  |  |
| Total | 23 | 180,456,144 |  |  |  |  |  |  |  |
|  |  |  |  |  |  |  |  |  |  |
| *Grouping Information Using the Tukey Method and 95% Confidence* | | | | |  |  |  |  |  |
| **Exposure Time*NPBI** | **N** | **Mean** | **Grouping** | | | | | |  |
| 15 Control | 3 | 8,303 | A |  |  |  |  |  |  |
| 30 Control | 3 | 7,213 |  | B |  |  |  |  |  |
| 15 Test | 3 | 6,963 |  | B |  |  |  |  |  |
| 45 Control | 3 | 5,492 |  |  | C |  |  |  |  |
| 30 Test | 3 | 3,910 |  |  |  | D |  |  |  |
| 60 Control | 3 | 3,160 |  |  |  | D |  |  |  |
| 45 Test | 3 | 1,575 |  |  |  |  | E |  |  |
| 60 Test | 3 | 1 |  |  |  |  |  | F |  |
| ***Means that do not share a letter are significantly different.* | | | | | | | | | |
|  |  |  |  |  |  |  |  |  |  |
| **SARS-CoV-2 Alpha variant @ 4,900 ions** | | |  |  |  |  |  |  |  |
| *Analysis of Variance* |  |  |  |  |  |  |  |  |  |
| **Source** | **DF** | **Adj SS** | **Adj MS** | **F-Value** | **P-Value** |  |  |  |  |
| Exposure Time | 1 | 63,089 | 63,089 | 173 | 0 |  |  |  |  |
| NPBI | 1 | 22,679 | 22,679 | 62 | 0 |  |  |  |  |
| Exposure Time*NPBI | 1 | 35 | 35 | 0 | 0.767 |  |  |  |  |
| Error | 8 | 2,924 | 366 |  |  |  |  |  |  |
| Total | 11 | 88,727 |  |  |  |  |  |  |  |
|  |  |  |  |  |  |  |  |  |  |
| *Grouping Information Using the Tukey Method and 95% Confidence* | | | | |  |  |  |  |  |
| **Exposure Time*NPBI** | **N** | **Mean** | **Grouping** | | | |  |  |  |
| 15 Control | 3 | 232 | A |  |  |  |  |  |  |
| 15 Test | 3 | 149 |  | B |  |  |  |  |  |
| 30 Control | 3 | 91 |  |  | C |  |  |  |  |
| 30 Test | 3 | 0 |  |  |  | D |  |  |  |
| ***Means that do not share a letter are significantly different.* | | | | | | | | | |
| **SARS-CoV-2 Alpha variant @ 12,000 ions** | | |  |  |  |  |  |  |  |
| *Analysis of Variance* |  |  |  |  |  |  |  |  |  |
| **Source** | **DF** | **Adj SS** | **Adj MS** | **F-Value** | **P-Value** |  |  |  |  |
| Exposure Time | 3 | 139,447 | 46,482 | 304 | 0 |  |  |  |  |
| NPBI | 1 | 114,305 | 114,305 | 747 | 0 |  |  |  |  |
| Exposure Time*NPBI | 3 | 6,836 | 2,279 | 15 | 0 |  |  |  |  |
| Error | 16 | 2,449 | 153 |  |  |  |  |  |  |
| Total | 23 | 263,037 |  |  |  |  |  |  |  |
|  |  |  |  |  |  |  |  |  |  |
| *Grouping Information Using the Tukey Method and 95% Confidence* | | | | |  |  |  |  |  |
| **Exposure Time*NPBI** | **N** | **Mean** | **Grouping** | | | | | |  |
| 10 Control | 3 | 312 | A |  |  |  |  |  |  |
| 20 Control | 3 | 232 |  | B |  |  |  |  |  |
| 10 Test | 3 | 225 |  | B | C |  |  |  |  |
| 25 Control | 3 | 193 |  |  | C |  |  |  |  |
| 30 Control | 3 | 134 |  |  |  | D |  |  |  |
| 20 Test | 3 | 84 |  |  |  |  | E |  |  |
| 25 Test | 3 | 11 |  |  |  |  |  | F |  |
| 30 Test | 3 | 0 |  |  |  |  |  | F |  |
| ***Means that do not share a letter are significantly different.* | | | | | | | | | |
| **SARS-CoV-2 Alpha variant @ 18,000 ions** | | |  |  |  |  |  |  |  |
| *Analysis of Variance* |  |  |  |  |  |  |  |  |  |
| **Source** | **DF** | **Adj SS** | **Adj MS** | **F-Value** | **P-Value** |  |  |  |  |
| Exposure Time | 3 | 85,832 | 28,611 | 691 | 0 |  |  |  |  |
| NPBI | 1 | 116,738 | 116,738 | 2,821 | 0 |  |  |  |  |
| Exposure Time*NPBI | 3 | 1,990 | 663 | 16 | 0 |  |  |  |  |
| Error | 16 | 662 | 41 |  |  |  |  |  |  |
| Total | 23 | 205,222 |  |  |  |  |  |  |  |
|  |  |  |  |  |  |  |  |  |  |
| *Grouping Information Using the Tukey Method and 95% Confidence* | | | | |  |  |  |  |  |
| **Exposure Time*NPBI** | **N** | **Mean** | **Grouping** | | | | | |  |
| 10 Control | 3 | 277 | A |  |  |  |  |  |  |
| 20 Control | 3 | 204 |  | B |  |  |  |  |  |
| 25 Control | 3 | 157 |  |  | C |  |  |  |  |
| 10 Test | 3 | 152 |  |  | C |  |  |  |  |
| 30 Control | 3 | 118 |  |  |  | D |  |  |  |
| 20 Test | 3 | 45 |  |  |  |  | E |  |  |
| 25 Test | 3 | 1 |  |  |  |  |  | F |  |
| 30 Test | 3 | 0 |  |  |  |  |  | F |  |
| ***Means that do not share a letter are significantly different.* | | | | | | | | | |

Table S10: Test for Equal Variances using Levene Method

| **Influenza A/Liter versus Exposure Time, NPBI** | | |  |  |
| --- | --- | --- | --- | --- |
| Null hypothesis | All variances are equal | |  |  |
| Alternative hypothesis | At least one variance is different | |  |  |
| Significance level | α = 0.05 |  |  |  |
| *95% Bonferroni Confidence Intervals for Standard Deviations* | | | |  |
| **Exposure Time** | **NPBI** | **N** | **StDev** | **CI** |
| 15 | Control | 3 | 316.125 | (0.0016552, 681887535) |
| 15 | Test | 3 | 268.284 | (0.0014047, 578693595) |
| 30 | Control | 3 | 240.82 | (0.0012609, 519452634) |
| 30 | Test | 3 | 298.436 | (0.0015626, 643731285) |
| 45 | Control | 3 | 142.106 | (0.0007440, 306524903) |
| 45 | Test | 3 | 171.906 | (0.0009001, 370805099) |
| 60 | Control | 3 | 744.005 | (0.0038955, 1604832808) |
| 60 | Test | 3 | 7.639 | (0.0000400, 16476999) |
| *Individual confidence level = 99.375%* | |  |  |  |
|  |  |  |  |  |
| **Method** | **Test Statistic** | **P-Value** |  |  |
| Multiple comparisons | — | 0 |  |  |
| Levene | 1.11 | 0.406 |  |  |
|  |  |  |  |  |
| **Influenza B/Liter versus Exposure Time, NPBI** | | |  |  |
| Null hypothesis | All variances are equal | |  |  |
| Alternative hypothesis | At least one variance is different | |  |  |
| Significance level | α = 0.05 |  |  |  |
| *95% Bonferroni Confidence Intervals for Standard Deviations* | | | |  |
| **Exposure Time** | **NPBI** | **N** | **StDev** | **CI** |
| 15 | Control | 3 | 31.53 | (0.0001651, 68010774) |
| 15 | Test | 3 | 25.8462 | (0.0001353, 55750759) |
| 30 | Control | 3 | 15.927 | (0.0000834, 34354927) |
| 30 | Test | 3 | 43.2317 | (0.0002264, 93251517) |
| 45 | Control | 3 | 15.9281 | (0.0000834, 34357095) |
| 45 | Test | 3 | 16.2118 | (0.0000849, 34969194) |
| 60 | Control | 3 | 10.4249 | (0.0000546, 22486686) |
| 60 | Test | 3 | 45.2105 | (0.0002367, 97519956) |
| *Individual confidence level = 99.375%* | |  |  |  |
|  |  |  |  |  |
| **Method** | **Test Statistic** | **P-Value** |  |  |
| Multiple comparisons | — | 0.554 |  |  |
| Levene | 0.45 | 0.854 |  |  |
|  |  |  |  |  |
| **RSV/Liter versus Exposure Time, NPBI** | |  |  |  |
| Null hypothesis | All variances are equal | |  |  |
| Alternative hypothesis | At least one variance is different | |  |  |
| Significance level | α = 0.05 |  |  |  |
| *95% Bonferroni Confidence Intervals for Standard Deviations* | | | |  |
| **Exposure Time** | **NPBI** | **N** | **StDev** | **CI** |
| 15 | Control | 3 | 31.612 | (0.0001655, 68187543) |
| 15 | Test | 3 | 43.4145 | (0.0002273, 93645819) |
| 30 | Control | 3 | 52.1364 | (0.0002730, 112459189) |
| 30 | Test | 3 | 62.358 | (0.0003265, 134507279) |
| 45 | Control | 3 | 87.4535 | (0.0004579, 188638815) |
| 45 | Test | 3 | 47.7852 | (0.0002502, 103073574) |
| 60 | Control | 3 | 59.817 | (0.0003132, 129026313) |
| 60 | Test | 3 | 14.3284 | (0.0000750, 30906614) |
| *Individual confidence level = 99.375%* | |  |  |  |
|  |  |  |  |  |
| **Method** | **Test Statistic** | **P-Value** |  |  |
| Multiple comparisons | — | 0.388 |  |  |
| Levene | 0.51 | 0.815 |  |  |
|  |  |  |  |  |
| **SARS-Delta/Liter versus Exposure Time, NPBI** | | |  |  |
| Null hypothesis | All variances are equal | |  |  |
| Alternative hypothesis | At least one variance is different | |  |  |
| Significance level | α = 0.05 |  |  |  |
| *95% Bonferroni Confidence Intervals for Standard Deviations* | | | |  |
| **Exposure Time** | **NPBI** | **N** | **StDev** | **CI** |
| 15 | Control | 3 | 177.725 | (0.0009305, 383355329) |
| 15 | Test | 3 | 113.778 | (0.0005957, 245422082) |
| 30 | Control | 3 | 297.564 | (0.0015580, 641851176) |
| 30 | Test | 3 | 267.288 | (0.0013995, 576544247) |
| 45 | Control | 3 | 378.727 | (0.0019830, 816920168) |
| 45 | Test | 3 | 348.187 | (0.0018231, 751046264) |
| 60 | Control | 3 | 325.163 | (0.0017025, 701383278) |
| 60 | Test | 3 | 0.317 | (0.0000017, 684832) |
| *Individual confidence level = 99.375%* | |  |  |  |
|  |  |  |  |  |
| **Method** | **Test Statistic** | **P-Value** |  |  |
| Multiple comparisons | — | 0 |  |  |
| Levene | 0.57 | 0.769 |  |  |
|  |  |  |  |  |
| **SARS@12K ions versus Exposure Time, NPBI** | | |  |  |
| Null hypothesis | All variances are equal | |  |  |
| Alternative hypothesis | At least one variance is different | |  |  |
| Significance level | α = 0.05 |  |  |  |
| *95% Bonferroni Confidence Intervals for Standard Deviations* | | | |  |
| **Exposure Time** | **NPBI** | **N** | **StDev** | **CI** |
| 10 | Control | 3 | 10.1281 | (0.0003991, 2488350) |
| 10 | Test | 3 | 19.8951 | (0.0007839, 4887981) |
| 20 | Control | 3 | 15.5388 | (0.0006123, 3817701) |
| 20 | Test | 3 | 10.4452 | (0.0004116, 2566265) |
| 25 | Control | 3 | 8.3547 | (0.0003292, 2052646) |
| 25 | Test | 3 | 8.6497 | (0.0003408, 2125121) |
| 30 | Control | 3 | 15.2037 | (0.0005991, 3735350) |
| 30 | Test | 3 | 0 | (*, *) |
| *Individual confidence level = 99.2857%* | |  |  |  |
|  |  |  |  |  |
| **Method** | **Test Statistic** | **P-Value** |  |  |
| Multiple comparisons | — | 0.885 |  |  |
| Levene | 0.3 | 0.925 |  |  |
| *Samples are omitted from the tests if their standard deviations are 0 or missing.* | | | | |
|  |  |  |  |  |
| **SARS@18K ions versus Exposure Time, NPBI** | | |  |  |
| Null hypothesis | All variances are equal | |  |  |
| Alternative hypothesis | At least one variance is different | |  |  |
| Significance level | α = 0.05 |  |  |  |
| *95% Bonferroni Confidence Intervals for Standard Deviations* | | | |  |
| **Exposure Time** | **NPBI** | **N** | **StDev** | **CI** |
| 10 | Control | 3 | 3.6621 | (0.0000192, 7899319) |
| 10 | Test | 3 | 6.1355 | (0.0000321, 13234483) |
| 20 | Control | 3 | 9.1107 | (0.0000477, 19651991) |
| 20 | Test | 3 | 7.1218 | (0.0000373, 15361875) |
| 25 | Control | 3 | 10.8441 | (0.0000568, 23390977) |
| 25 | Test | 3 | 0.4599 | (0.0000024, 992107) |
| 30 | Control | 3 | 5.3318 | (0.0000279, 11500745) |
| 30 | Test | 3 | 0.0041 | (0.0000000, 8835) |
| *Individual confidence level = 99.375%* | |  |  |  |
|  |  |  |  |  |
| **Method** | **Test Statistic** | **P-Value** |  |  |
| Multiple comparisons | — | 0 |  |  |
| Levene | 1.26 | 0.328 |  |  |
|  |  |  |  |  |
| **SARS@5k ions versus Exposure Time, NPBI** | | |  |  |
| Null hypothesis | All variances are equal | |  |  |
| Alternative hypothesis | At least one variance is different | |  |  |
| Significance level | α = 0.05 |  |  |  |
| *95% Bonferroni Confidence Intervals for Standard Deviations* | | | |  |
| **Exposure Time** | **NPBI** | **N** | **StDev** | **CI** |
| 15 | Control | 3 | 15.0874 | (0.057721, 23553.7) |
| 15 | Test | 3 | 18.924 | (0.072399, 29543.2) |
| 30 | Control | 3 | 29.6005 | (0.113245, 46210.7) |
| 30 | Test | 3 | 0.3556 | (0.001361, 555.2) |
| *Individual confidence level = 98.75%* | |  |  |  |
|  |  |  |  |  |
| **Method** | **Test Statistic** | **P-Value** |  |  |
| Multiple comparisons | — | 0 |  |  |
| Levene | 0.86 | 0.501 |  |  |
